# Characterization of a New Biocomposite Based on Bioactive Compounds from *Ganoderma lucidum* and Jellyfish Collagen Destined for In Vitro Evaluation of Antitumor Effects in the Oral Cavity

**DOI:** 10.3390/ph19010108

**Published:** 2026-01-07

**Authors:** Carolina Pascale, Alexandru Burcea, Claudia Florina Bogdan-Andreescu, Emin Cadar, Antoanela Popescu, Ticuta Negreanu-Pirjol, Florica Busuricu, Ana-Maria Pesterau, Adrian Cosmin Rosca, Rodica Sirbu

**Affiliations:** 1 Organizing Institution for Doctoral University Studies of “Carol Davila”, Faculty of Pharmacy, “Carol Davila” University of Medicine and Pharmacy Bucharest, 020021 Bucharest, Romania; carolina.pascale@drd.umfcd.ro (C.P.); ana-maria.pesterau@drd.umfcd.ro (A.-M.P.); sirbu_27@yahoo.com (R.S.); 2 Faculty of Dental Medicine, Department of Speciality Disciplines, “Titu Maiorescu” University, 031593 Bucharest, Romania; alexandru.burcea@helpdent.ro (A.B.); claudia.andreescu@prof.utm.ro (C.F.B.-A.); 3 Faculty of Pharmacy, “Ovidius” University of Constanta, 900470 Constanta, Romania; ticuta.negreanu@univ-ovidius.ro (T.N.-P.); busuricuflori@yahoo.com (F.B.); cosmin.rosca@univ-ovidius.ro (A.C.R.)

**Keywords:** oral squamous cell carcinoma, *Ganoderma lucidum*, marine collagen, biocomposite hydrogel, antioxidant and antimicrobial activity

## Abstract

**Background/Objectives:** Oral squamous cell carcinoma (OSCC) remains a major therapeutic challenge due to treatment-related toxicity and impaired oral tissue regeneration. This study aimed to develop and characterize a novel biocomposite based on bioactive compounds from *Ganoderma lucidum* incorporated into marine collagen derived from *Rhizostoma pulmo* and to evaluate its physicochemical properties, antioxidant and antimicrobial activities, and in vitro antitumor potential in the oral cavity. **Methods:** Hydroalcoholic extracts of *G. lucidum* and pepsin-soluble collagen peptides from *R. pulmo* jellyfish were prepared and combined to obtain two hydrogel biocomposites with different component ratios. Chemical and structural characterization was performed using HPLC-DAD, SDS-PAGE, FT-IR, circular dichroism, and spectrophotometric assays. Antioxidant activity was assessed by DPPH radical scavenging and reducing power assays, while antimicrobial activity was evaluated against oral pathogens using diffusion and MIC methods. In vitro biological activity was investigated using MTT viability and scratch migration assays on human OSCC cell lines (SCC-9 and HSC-3). **Results:** The biocomposites preserved the structural integrity of type I collagen and incorporated polysaccharides and polyphenols from *G. lucidum*. The combined formulations showed enhanced antioxidant and antimicrobial activities compared with collagen alone. In vitro assays demonstrated dose- and time-dependent reductions in OSCC cell viability and delayed cell migration, with effects comparable to those of *G. lucidum* extract. **Conclusions:** The *G. lucidum*–*R. pulmo* biocomposite exhibits favorable physicochemical properties and demonstrates antioxidant, antimicrobial, and in vitro antitumor activity. These findings support its potential as a multifunctional biomaterial for further investigation as an adjunct approach in oral cancer-related applications.

## 1. Introduction

Globally, oral diseases are among the most prevalent health conditions and represent a major public health concern, significantly reducing individuals’ quality of life, as highlighted by Peres et al. (2019) [1]. Among these, oral cancer is a particularly serious disease encompassing a wide spectrum of neoplasms located in the oral cavity, pharynx, and salivary glands. The term is largely synonymous with oral squamous cell carcinoma (OSCC). Abed et al. (2019), Warnakulasuriya et al. (2020), and Sung et al. (2021) have described various aspects of OSCC, which is the most common form of oral cancer among oral tumors [2,3,4]. Studies by Gaździcka et al. (2020), Wan et al. (2020), Bugshan et al. (2020), and Jung et al. (2020) indicate that OSCC accounts for more than 90% of head and neck squamous cell carcinomas (HNSCCs), with a five-year survival rate ranging between 40% and 50%. It is also considered the sixth most common type of cancer worldwide [5,6,7,8]. According to Sato et al. (2019) and Ghosh et al. (2019), current medical practice typically treats OSCC using a combination of conventional therapies, such as surgery, radiotherapy, and chemotherapy, employing agents including cisplatin, 5-fluorouracil, and docetaxel, among others [9,10]. However, Ghosh et al. (2019) reported that certain chemotherapeutic drugs, such as cisplatin or (SP-4-2)-diamminedichloroplatinum (II), although widely used in the treatment of various cancers, are highly toxic and present significant disadvantages in clinical applications due to their adverse side effects and the development of drug resistance [10]. Cheng et al. (2021) further evidenced mechanisms underlying cisplatin resistance in oral squamous cell carcinoma (OSCC) [11]. Ezzat et al. (2021) noted that radiotherapy may also cause several adverse events, including xerostomia, dental caries, oral mucositis, trismus, and osteoradionecrosis [12]. Similarly, Bharadwaj et al. (2019) and Zhang et al. (2020) analyzed the undesirable side effects associated with conventional chemotherapy, such as anemia, neutropenia, vomiting, oral inflammation and infections, neurotoxicity, hepatotoxicity, and nephrotoxicity. These treatments can also impair vital oral and facial functions, including speech, breathing, taste, smell, chewing, swallowing, and overall facial appearance [13,14]. The high costs, low survival rates, and slow progress in oral cancer therapy research have also been emphasized by other researchers, including Wong et al. (2018) [15]. Khalil et al. (2017) identified epidermal growth factor receptors (EGFRs) as a potential therapeutic target in oral cavity cancer. However, their study also reported that the use of EGFR inhibitors can cause multiple adverse effects, such as skin reactions, nausea, and diarrhea. Moreover, EGFR inhibition may enhance cisplatin-induced apoptosis in OSCC cells, but paradoxically, it can also contribute to increased tumor cell proliferation and metastasis [16]. Efforts to minimize the toxic effects experienced by patients undergoing conventional treatments have prompted the development of new therapeutic strategies for oral cancer. In this context, herbal medicines have gained attention for their potential anticancer properties. Imram et al. (2019), Nazhvani et al. (2020), and Ma et al. (2020) conducted studies exploring the use of herbal medicines in anticancer therapy [17,18,19]. More recently, Billi et al. (2025) investigated the biological potential of cannabinol in human oral squamous cell carcinoma cells [20]. The remarkable anticancer properties of *Ganoderma lucidum* biocomponents have been recognized in studies by Cadar et al. (2023) and Toson et al. (2025) [21,22]. Kumar et al. (2019) reported that in Chinese medicine, *G. lucidum* have been used to treat a wide range of acute and chronic diseases, including cancer [23]. According to Bhat et al. (2021), these therapeutic effects are attributed to various classes of bioactive compounds distributed throughout the mushroom [24]. Among these, polysaccharides exhibit antitumor, immunomodulatory, antioxidant, and antiangiogenic activities. Terpenes, particularly triterpenoids demonstrate antiproliferative, antiangiogenic, anti-inflammatory, and antioxidant effects. Proteins, amino acids, and polyphenols primarily contribute antioxidant and antitumor activities, while vitamins, minerals, sterols, and fibers are also known for their anticancer potential. It is noteworthy that similar bioactive compounds are also present in other marine resources, as reported by Sîrbu et al. (2019), Cadar et al. (2023), and Cadar et al. (2025) [25,26,27,28]. Kim et al. (2024), Haleem et al. (2024), and Camargo et al. (2022) studied the pharmacological actions of *G. lucidum* in the oral cavity [29,30,31]. Hsu et al. (2021) isolated a water-soluble, glucose-rich polysaccharide (WSG) from *Ganoderma lucidum* and tested its effects on human tongue cancer cell lines (SAS and HSC3). Their results demonstrated that WSG significantly reduced cell viability and colony formation in these cell lines [32]. Zeng et al. (2020) further demonstrated advances in OSCC treatment through modulation of the miR-188/BCL9/β-catenin pathway [33].

Another material of interest in our study is collagen, a biological macromolecule with a triple-helix structure. Xu et al. (2021) described the different types of collagen and emphasized its value as a biomaterial due to its accessibility, biocompatibility and biodegradability [34]. In recent years, Cherim et al. (2019), Cadar et al. (2023) and Pesterau et al. (2025) have shown that collagen extracted from marine sources has become widely used compared to bovine and porcine collagen due to the possibility of transmitting diseases such as foot-and-mouth disease, bovine spongiform encephalopathy, or infectious encephalopathies [35,36,37]. Collagen extraction from marine resources must be performed using carefully controlled methods, as demonstrated by Sîrbu et al. (2019) and Cadar et al. (2024) [38,39]. An essential requirement, emphasized by Cherim et al. (2017) and Cadar et al. (2018, 2019), is that the marine environment used as a source for collagen extraction must be clean and free from pollutants [40,41,42]. The antitumor and antioxidant activities of marine collagen have been evidenced by several authors, including Mizarpour et al. (2020), Yaghoubzadeh et al. (2019), Wali et al. (2019), and Rahman et al. (2020) [43,44,45,46]. Marine collagen is particularly valuable because of its role in promoting oral mucosa regeneration, enhancing cell viability and migration, exhibiting antitumor potential and demonstrating antimicrobial effects against common oral pathogens. Based on the above observations, we propose new perspectives for the development of combined biomaterials that synergistically improving the anticancer actions of bioactive compounds from *G. lucidum* (rich in polysaccharides, triterpenes, and phenolic compounds) with collagen compounds from *R. pulmo.* These biomaterials are intended to promote oral health through demonstrated in vitro antitumor activities, antioxidant and antimicrobial.

Due to the limited data available in the specialized literature regarding the antitumor activity of composites based on fungal extracts and marine collagen derived from jellyfish, particularly on oral tumor cell lines, the aim of the present study was to identify and characterize the biocomponents of *Ganoderma lucidum* and jellyfish collagen, as well as to evaluate the antioxidant, antimicrobial, and in vitro anticancer activities of a novel composite obtained by combining these two natural resources. As highlighted in previous studies, oral squamous cell carcinoma (OSCC) remains a major clinical challenge worldwide, primarily due to its high recurrence rate and the limitations of current therapeutic approaches [3,4]. Conventional anticancer agents are often associated with significant drawbacks, including systemic toxicity, limited selectivity toward tumor tissue, and adverse side effects, which have driven the search for alternative or complementary therapeutic strategies. In this context, the development and evaluation of an innovative natural biocomposite with potential antitumor activity represent a promising adjuvant approach.

More specifically, a series of preliminary investigations were conducted on the newly developed biocomposite, focusing on its rheological characteristics, antioxidant and antimicrobial properties, and in vitro antitumor potential. The composite was obtained by incorporating hydroalcoholic extracts of *G. lucidum* into a collagen matrix extracted from *Rhizostoma pulmo* jellyfish harvested from the Black Sea. In addition, the study comparatively evaluated the antioxidant and antimicrobial efficacy, as well as the rheological behavior, of the composite relative to its individual biocomponents. Preliminary in vitro experiments performed on OSCC cell lines were designed to assess the capacity of this innovative biocomposite to inhibit cell migration and reduce tumor cell viability, thereby demonstrating its biological activity rather than claiming immediate clinical selectivity or therapeutic applicability.

Although the biological activities of *Ganoderma lucidum* extracts and marine-derived collagen have each been reported previously, studies that integrate these two natural resources into a single multifunctional biomaterial remain limited. In particular, there is a lack of systematic investigations addressing the combined physicochemical, rheological, antimicrobial, antioxidant, and anticancer properties of fungal–marine biocomposites designed for applications in the oral cavity.

The present study addresses this gap by developing and characterizing a novel hydrogel biocomposite that incorporates bioactive compounds from *G. lucidum* into a collagen matrix derived from the jellyfish *Rhizostoma pulmo*. The originality of this work lies not in the discovery of new individual bioactivities but in the formulation of a composite material and the evaluation of potential synergistic interactions between its components, with particular emphasis on in vitro antitumor activity against oral squamous cell carcinoma (OSCC) cell lines and antimicrobial activity against oral pathogens. This integrated approach aims to provide a foundation for further studies on multifunctional biomaterials intended for oral health applications.

## 2. Results

**Figure 1:**
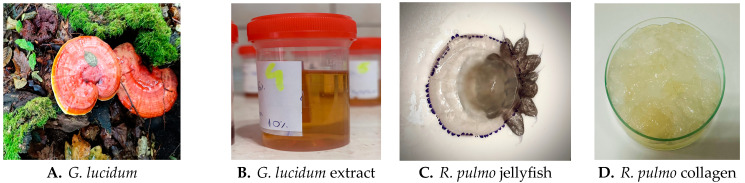
Raw materials used to formulate a new biocomposite based on *G. lucidum* extract and collagen obtained from *R. pulmo* [47].

The pharmaceutical formulations developed in this study are intended for the treatment of conditions associated with oral squamous cell carcinoma (OSCC). These formulas are designed as hydrogels composed of hydroalcoholic extracts from *G. lucidum* and marine collagen extracted from *R. pulmo*. In the initial phase, both species were analyzed separately to characterize their chemical and biological properties. Subsequently, new biocomposites combining extracts from *G. lucidum* and *R. pulmo* were developed and evaluated for their potential use in OSCC therapy within the oral cavity. Figure 1 presents images of the *G. lucidum* mushroom and the collagen hydrolysate obtained from *R. pulmo* jellyfish collected from the Black Sea.

### 2.1. Chemical Profiling of Ganoderma lucidum Ethanolic Extract and Rhizostoma pulmo Collagen Hydrolysate

The *G. lucidum* extract was obtained with a yield of 90% by maceration in 70% ethyl alcohol. The collagen from *R. pulmo* was obtained with a yield of 55.6% by extraction using the PSC method (pepsin-soluble collagen). Table 1 shows the approximate composition of the extract from the *G. lucidum* mushroom and the collagen from *R. pulmo.* The results are given as percentages relative to dry weight (DW). The moisture content for the *G. lucidum* extract, was 11.98 ± 4.34%, consistent with the results reported by Rahman et al. (2020) and Gharib et al. (2022). The hydrogel extract (JPC) from *R. pulmo* exhibited a moisture content of 13.1 ± 0.1% [46,48]. The ash content was also determined for each material analyzed. Among the key results, the polysaccharide content of the *G. lucidum* extract was notably high, at 42.80 ± 5.53%, aligning with previously reported data by Rahman et al. (2020), Gharib et al. (2022), and Taofiq et al. (2017) [46,48,49]. In contrast, the collagen extracts from *R. pulmo* showed a relatively low polysaccharide content of 1.25 ± 0.30% (W) and 0.65 ± 0.25% (G). The protein content of *G. lucidum* extracts was relatively low, at 7.49 ± 0.56% of dry weight (DW), consistent with previous reports by Gharib et al. (2022), Taofiq et al. (2017) and El Sheikha et al. (2022) [48,49,50]. In contrast, extracts from the jellyfish *R. pulmo* exhibited a much higher protein content of 59.48 ± 0.55%, with protein, primarily collagen, being the predominant component (55.5 ± 0.6%). Similar values have been reported by Pesterau et al. (2024), Leone et al. (2015), and D’Ambra et al. (2015, 2022) [37,51,52,53]. The lipid content of *G. lucidum* extracts was 2.83 ± 0.18%, comparable to the data reported by Rahman et al. (2020) and Taofiq et al. (2017) [46,49]. El Sheikha et al. (2022) also presented comparable data for lipid content [50]. The whole-body lipid content of *R. pulmo* was 5.2 ± 0.79% comparable to the data of D’Ambra et al. (2022) [53]. Additionally, the total dietary fiber content of *G. lucidum* extracts was analyzed.

The biocomponents of interest are polysaccharides, proteins, and polyphenol content, which support the antioxidant and antimicrobial activities of the biomaterials studied.

### 2.2. Monosaccharide Types of Polysaccharide Content of G. lucidum

The results obtained by the HPLC-DAD method used to quantify the basic units of monosaccharides in the polysaccharides identified in *G. lucidum* are presented in Table 2.

**Figure 2:**
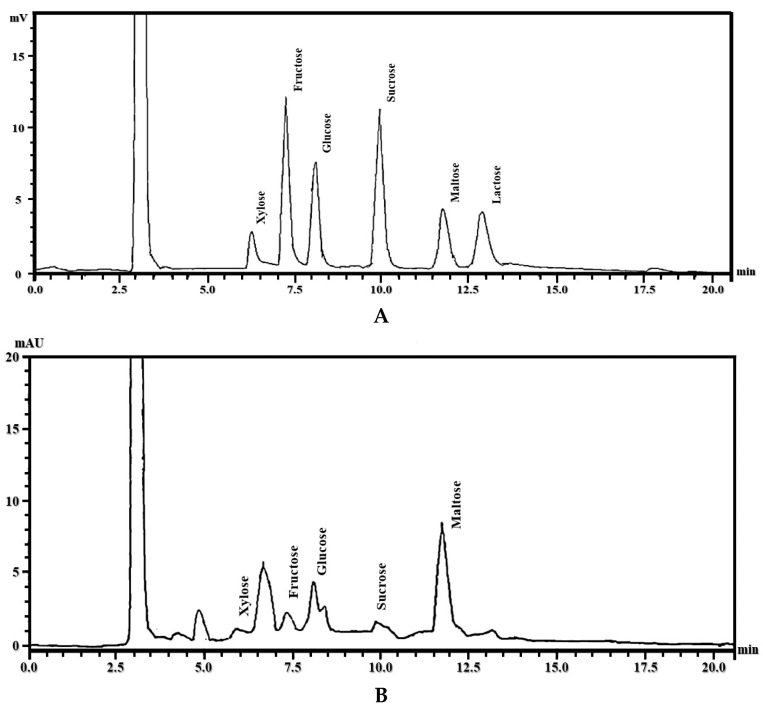
Chromatograms obtained in HPLC analysis: (**A**) standards for the analyzed monosaccharides; (**B**) results for monosaccharides in the extract from *G. lucidum*.

The results show that maltose was the main monosaccharide, followed by fructose, glucose, sucrose, and xylose. Figure 2 shows the chromatograms obtained for the monosaccharide standards and the ethanolic extract of *G. lucidum.*

### 2.3. Rhizostoma pulmo Collagen Characterization

#### 2.3.1. SDS-PAGE Analysis

**Figure 3:**
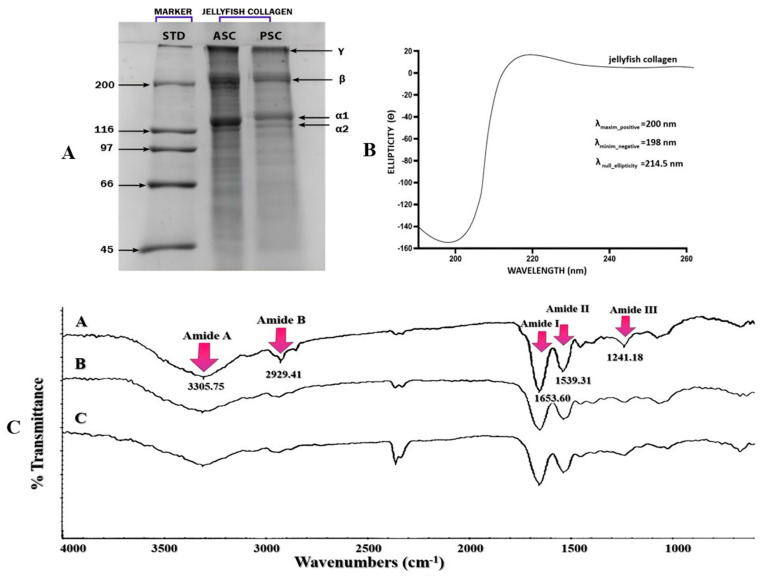
Physicochemical analysis on the jellyfish collagen: (**A**). SDS-PAGE analysis of collagen from *R. pulmo*; (**B**). circular dichroism spectra of *R. pulmo* collagen; (**C**). FT-IR spectra for *R. pulmo* collagen [37].

The protein content of *R. pulmo* jellyfish consists primarily of collagen and collagen peptides. To characterize the structure of collagen SDS-PAGE analysis was performed. The electrophoretic profiles of both preparations were very similar, confirm that the isolated protein is type I collagen. Type I bovine collagen was used as a standard, and the α-chain bands were observed in the 110–150 kDa range. In Figure 3A, bands of the β and γ chains are also detected above 200 kDa.

#### 2.3.2. Circular Dichroism Spectral Analysis

The secondary structure of collagen obtained from the jellyfish *R. pulmo* was determined by circular dichroism (CD) spectroscopy, and the results obtained are illustrated in Figure 3B. The spectrum showed a positive ellipticity band around 200 nm and a minimum at 198 nm. The signals support the maintenance of the triple-helix configuration characteristic of collagen.

#### 2.3.3. FT-IR Analysis

Figure 3C shows the FT-IR spectrum obtained for collagen extracted from *R. pulmo,* tracking the variation in transmittance as a function of wavelength. Spectral analysis confirms type I collagen with a triple helix structure.

FT-IR spectroscopy can provide information on collagen structure based on the presence and intensity of distinct peaks corresponding to certain types of amides, such as A/B and amide bonds I, II, and III, which are essential for triple helix formation, as shown Belbachir et al. (2009) and Riaz et al. (2018) [54,55]. The presence of proline is indicated by signals at 1444 cm^−1^ and 1334 cm^−1^. Additionally, the amide II peak observed in the range 1533–1537 cm^−1^ further confirms the preservation of the collagen triple-helix structure. The signal at 3283 cm^−1^ is attributed to N–H vibrations (amide A), while the signal at 2934 cm^−1^ is attributed to CH_2_ groups (amide B). The signal at 1647 cm^−1^, 1534 cm^−1^, and 1241 cm^−1^ are attributed to amides I, II, and III, associated with C=O, C–N, and N–H vibrations, respectively. Our results are comparable to data in the literature. The signal at 1.241.18 cm^−1^ (amide III) confirms the existence of an intact triple helical structure, demonstrating the quality and stability of collagen extracted from *R. pulmo*, as also shown by Derkus et al. (2016) [56]. At a signal at 1.539.31 cm^−1^, amide II was present, and it can be stated that hydrogen bonds exist, in accordance with other previous studies conducted by Riaz et al. (2018) [55]. Amide I was at 1.651 cm^−1^ in *R. pulmo*, and the signal of amide II was 1539 cm^−1^ in accordance with studies conducted by Cadar et al. (2024) and Riaz et al. (2018) [39,55]. Amide III showed a signal frequency at 1241.18 cm^−1^ close to 1238 cm^−1^ in *R. pulmo* in accordance with studies conducted by Derkus et al. (2016) [56].

Table 3 presents the values obtained by us compared with data from the literature.

### 2.4. Amino Acid Content of R. pulmo

The results for the amino acid compositions from whole body of *R. pulmo* jellyfish harvested from the Romanian Black Sea coast (reported as residues per 1000 residues) are presented in Table 4. From this analysis, it is evident that collagen extracts from *R. pulmo* provide an important contribution of amino acids, and our results are comparable with the literature data of James et al. (2023) [57].

### 2.5. Polyphenolic Content

#### 2.5.1. Total Phenolic and Flavonoid Content

Table 5 shows the results for total phenolic content (TPC) and total flavonoid content (TFC). Both TPC and TFC were detected in *G. lucidum* extracts, whereas only TPC was found in the collagen extracts and at substantially lower levels. These findings are consistent with previous reports, with Rahman et al. (2020) documenting similar TPC values in *G. lucidum* and Leone et al. (2015) reporting comparable TPC in *R. pulmo* collagen extracts [46,51]. The TFC was evaluated exclusively in *G. lucidum* and compared with data from Gąsecka et al. (2016) [58]. The presence of polyphenols, together with polysaccharides, is important for enhancing antioxidant activity.

#### 2.5.2. Individual Phenolic Acids

Table 6 shows the results for individual polyphenols obtained for the fruiting body of *G. lucidum* reported as dry weight (DW) and those obtained for the whole body of *R. pulmo* reported as wet weight (WW). We found that in the extract of *G. lucidum,* the most abundant polyphenol was gallic acid, followed by kaempferol, ellagic acid, and 3-methylgallic acid. In *R. pulmo*, only gallic, syringic, and caftaric acids were identified.

### 2.6. Characterization of the New Biocomposite Obtained

The formulation of the new biocomposite is based on an extract obtained from the *Ganoderma lucidum* mushroom and collagen peptides from the *Rhizostoma pulmo* jellyfish.

#### 2.6.1. Macroscopic Study of GL-JPC Composite

To develop the biocomposite composed of *G. lucidum* (GL) extract and jellyfish collagen peptides (JPCs), a hydroalcoholic extract of GL was incorporated into a collagen matrix derived from JPC. Two types of biocomposites were formulated:

- **Extract E1 (GL–JPC):** prepared with a composition of 3/5 (*v*/*v*) 10% GL ethanolic extract and 2/5 (*v*/*v*) JPC collagenic hydrogel extract.
- **Extract E2 (GL–JPC):** prepared with a composition of 4/5 (*v*/*v*) 10% GL ethanolic extract and 1/5 (*v*/*v*) JPC collagenic hydrogel extract.

The macroscopic characterization, including the organoleptic properties of the individual components and of the newly obtained formulations, was presented in Table 7.

For both formulations, freeze-dried collagen was used as the structural matrix, while the *G. lucidum* extract was incorporated in liquid form. The organoleptic evaluation of the GL–JPC composites revealed a distinct color variation correlated with the concentration of the fungal extract, shifting from yellowish-white to yellowish-brown as the proportion of *G. lucidum* increased. Specifically, the *G. lucidum* extract was used in liquid state, while the collagen obtained from *R. pulmo* jellyfish was used in freeze-dried powder form.

#### 2.6.2. Microscopic Study of GL-JPC Composite

**Figure 4:**
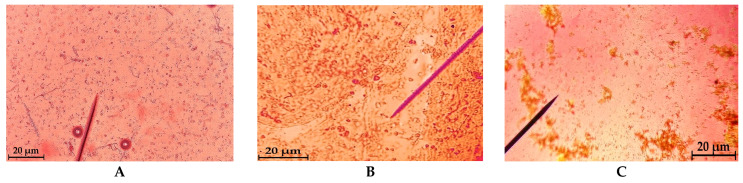
Microscopic images of the studied materials: (**A**) Image of *G. lucidum* extract; (**B**) Image of Collagen extract; (**C**) Image of biocomposite E1 (GL-JPC). Both were stained with hematoxylin and read at mixing time 0 h.

The microscopic study was conducted to directly observe the structural organization of the two primary components and to evaluate their interaction within the newly formulated biocomposite. Microscopic images of the extracts obtained from both studied species are presented in Figure 4. Following the incorporation of the two compounds, the resulting material exhibited a viscous and gelatinous texture. A gradual change in color was observed, ranging from light yellow in formulation E1 to light brown in formulation E2, corresponding to the increasing proportion of *G. lucidum* extract.

Figure 4 shows the microscopic analysis of the studied materials, namely, in Figure 4A, the *G. lucidum* extract shows fine, uniformly distributed aggregates, indicating a homogeneous extract; Figure 4B shows the collagen extract from the *R. pulmo* jellyfish shows the fibrillar structure characteristic of marine collagen; Figure 4C shows a microscopic image of the E1 (GL–JPC) biocomposite that illustrates the interaction between the fungal extract and marine collagen and highlights the initial stages of structural integration during biocomposite formation.

#### 2.6.3. Rheological Study of the Newly Obtained Biocomposites

**Figure 5:**
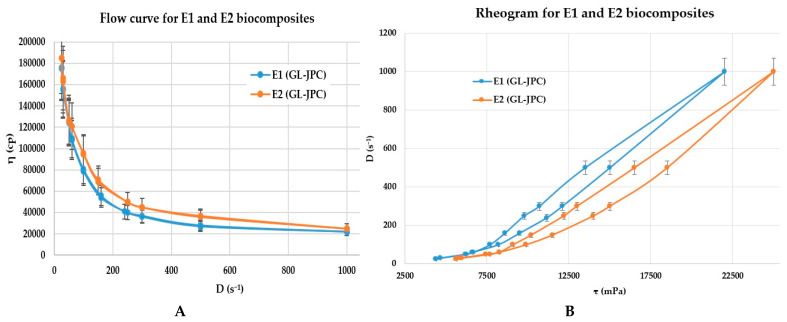
Flow curve of *G. lucidum* extract and jellyfish collagen formulation E1 and E2 based GL-JPC (**A**) and rheogram for the newly formulated E1 and E2 (**B**). Data are expressed as mean ± SD (*n* = 3).

To construct the flow curve (Figure 5A) and the rheogram (Figure 5B), the parameters described above were applied to the newly formulated hydrogels composed of *G. lucidum* ethanolic extract (10%) (GL) and jellyfish collagen peptides (JPCs) obtained from *R. pulmo*.

According to one-way Anova analysis, no statistical significant differences were observed between analyzed samples (*p* > 0.05, for *n* = 3), which suggests that both biocomposites induce similar effects under the same experimental conditions. In Figure 5, the data are presented as averages of three successive measurements with ±Standard Deviation. The rheological profile of both formulations demonstrates pseudoplastic (shear-thinning) behavior across the applied shear rate range, confirming a non-Newtonian response typical of hydrogel systems. Both graphs—shear stress versus shear rate—exhibited a hysteresis loop between the ascending and descending curves, indicating the thixotropic nature of the two composites, E1 and E2. The incorporation of GL extract at different concentrations into the JPC collagen matrix resulted in viscous arrays with distinct viscoelastic characteristics. Similar rheological characteristics for collagen-based preparations have previously been described by Cherim et al. (2019) and Pesterau et al. (2025) [35,37].

The E1 (GL–JPC) biocomposite exhibited higher viscosity, whereas the E2 (GL–JPC) formulation displayed slightly lower viscosity.

### 2.7. Antimicrobial Activity

**Figure 6:**
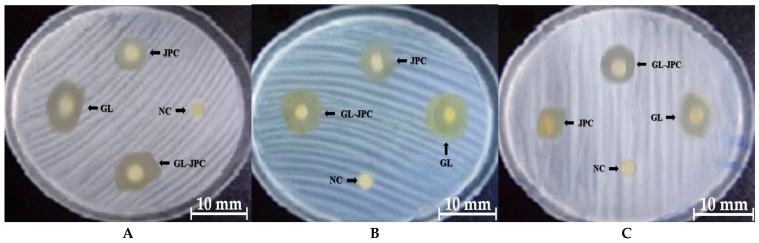
(**A**) Antimicrobial activity of GL, JPC, and E2 (GL-JPC) versus, (**B**) Antimicrobial, (**C**) Antimicrobial activity of GL, JPC, and E2 (GL-JPC) versus *C. albicans*; NC-negative control activity of E2 (GL-JPC), JPC, and GL against *S. mutans;* NC-negative control, *S. aureus*; NC-negative control.

The results of antimicrobial activity of the JPC collagen extract, GL ethanolic extract, and the newly developed E2 (GL–JPC) formulation were assessed against common oral pathogens: *S. mutans*, *S. aureus,* and *C. albicans*. The results are illustrated in Figure 6: *S. aureus* (Figure 6A), *S. mutans* (Figure 6B), and *C. albicans* (Figure 6C), with ethanol used as the negative control (NC).

A more extensive study in which antimicrobial activity was evaluated against the following pathogens: Gram-positive bacteria, *S. aureus* (ATCC 25923) and *S. mutans* (ATCC 25175), as well as the fungal strain *C. albicans (ATCC 10231).* The Gram-negative bacteria studied were *E. coli* (ATCC 25922), *P. aeruginosa* (ATCC 27853), and *P. mirabilis* (ATCC 25933).

**Figure 7:**
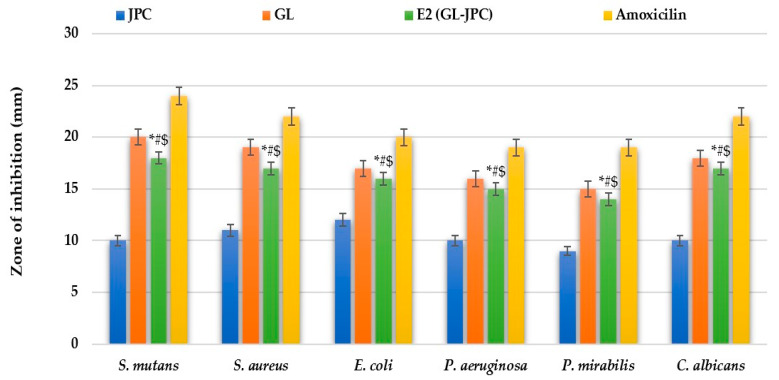
Antimicrobial activity of *G. lucidum* extract (orange), *R. pulmo* collagen (blue), E2 (GL-JPC) composite (green), and Amoxicillin, the control (yellow). Each result represents the mean ± SD for *n* = 3; * *p* < 0.01 for GL-JPC compared with JPC; # *p* < 0.05 for GL-JCP compared with GL and $ *p* < 0.05 for GL-JCP compared with Amoxicillin.

The E2 (GL–JPC) formulation was selected as the representative composite to compare the antimicrobial activity of the combined material with that of its individual GL and JPC components. Figure 7 presents the antimicrobial activity results expressed as inhibition zone diameters (mm), determined using the diffusimetric method. This method was applied to the ethanolic extract of *G. lucidum*, collagen peptides extracted from *R. pulmo*, the newly formulated biocomposite E2 (GL–JPC), and amoxicillin (used as a reference antibiotic). Statistical analysis using ANOVA and *t*-tests revealed significant differences in the inhibition zone diameters among the tested samples and amoxicillin: *p* < 0.01 (marked with *) for E2 (GL–JPC) compared to JPC; *p* < 0.05 (marked with #) for E2 (GL–JPC) compared to GL; and *p* < 0.05 (marked with $) for E2 (GL–JPC) compared to amoxicillin.

Table 8 shows the Minimum Inhibitory Concentration (MIC) values for the samples tested against the analyzed pathogens. The MIC values range from 25 µg/mL to 86.5 µg/mL.

For collagen peptides, our results are similar to those reported by Pesterau et al. (2025) and are consistent with the findings of these analyses [37].

An interesting finding of the antimicrobial evaluation was the enhanced inhibitory activity of the *G. lucidum*-*R. pulmo* biocomposite against *Staphylococcus aureus* compared with the individual components. This selective synergistic effect was not observed for other tested microorganisms, including *Streptococcus mutans* or *Candida albicans*. A plausible explanation may involve differences in cell wall structure and membrane composition among microbial species.

Polyphenols and triterpenoids present in *G. lucidum* extracts are known to disrupt bacterial membranes and interfere with intracellular targets, while collagen-derived peptides may contribute to improved adhesion or localized retention of bioactive compounds at the microbial surface. The combination of these mechanisms may enhance susceptibility in *S. aureus*, a Gram-positive bacterium with a thick but relatively permeable peptidoglycan layer. In contrast, differences in fungal cell wall composition or bacterial membrane architecture may limit similar synergistic effects in other strains. These hypotheses require further investigation through targeted mechanistic and repeated antimicrobial studies.

### 2.8. Antioxidant Activity

#### 2.8.1. DPPH Assay

**Figure 8:**
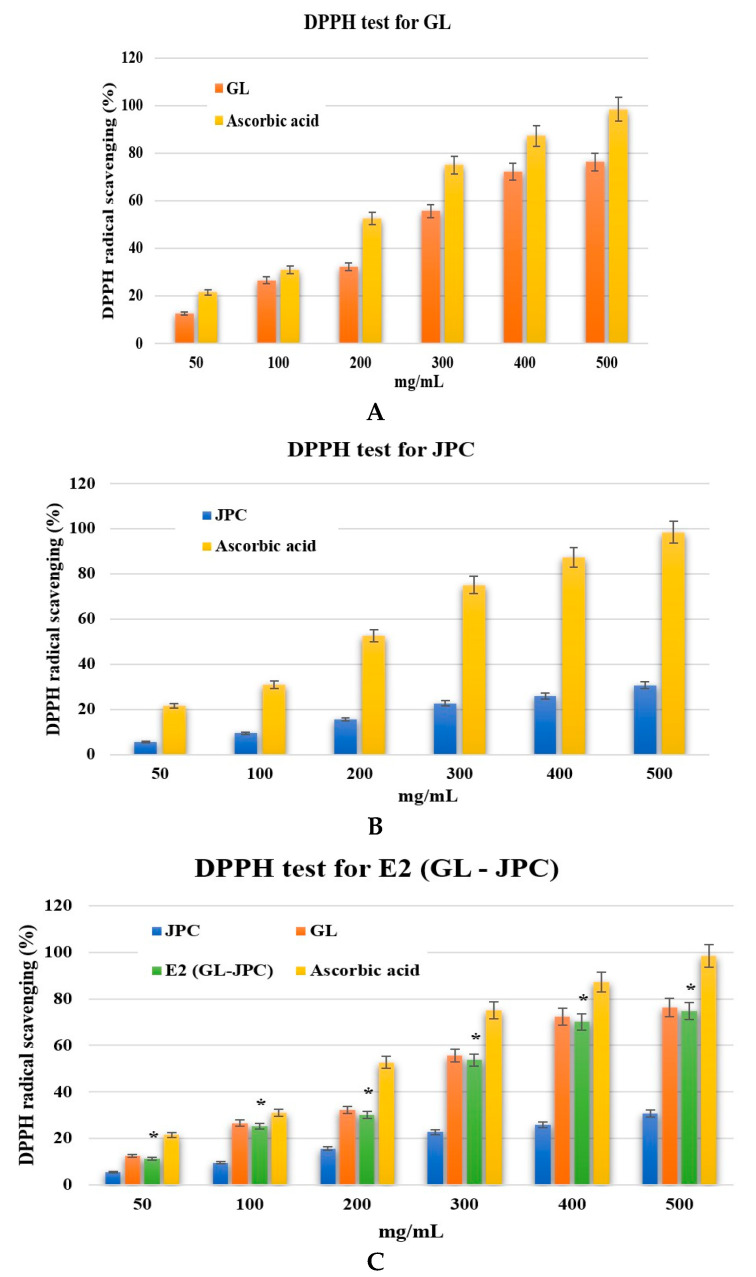
The results of DPPH test for *G. lucidum*–GL (**A**), *R. pulmo* collagen–JPC (**B**)**,** and newly formulated biocomposite compared to (**C**) GL-JPC with standard antioxidant (ascorbic acid). Results represents the mean ± SD for *n* = 3; * *p* < 0.05 for E2 compared with JPC.

The antioxidant capacity was evaluated using the DPPH assay, and the results are illustrated in Figure 8. The DPPH radical scavenging activity of the ethanolic extract of *G. lucidum* (GL) is presented in Figure 8A, while Figure 8B shows the antioxidant activity of collagen extracted from the jellyfish *R. pulmo* (JPC). Figure 8C compares the results of antioxidant capacity of the individual compounds (GL and JPC) with the newly developed GL–JPC biocomposite.

As shown in Figure 8, both the GL extract and the GL–JPC biocomposite exhibited significantly higher scavenging activity compared to JPC alone. When benchmarked against the standard antioxidant, ascorbic acid, the GL–JPC biocomposite demonstrated comparable activity, indicating a strong antioxidant potential. These results suggest that the combination of *G. lucidum* extract and *R. pulmo* collagen results in a synergistic enhancement of antioxidant properties, attributable to the complementary bioactive compounds present in the biocomposite.

The improved antioxidant capacity can be attributed to the high content of polysaccharides, polyphenols, and terpenoids present in the *G. lucidum* (GL) extract, which are considerably more abundant than in the *R. pulmo* collagen peptide (JPC) extract. The IC_50_ value of ascorbic acid, used as a reference standard, was 187.82 mg/mL. For the newly formulated biocomposite E2 (GL–JPC), the IC_50_ value was 231.44 mg/mL, while the values for *G. lucidum* and JPC were 225.74 mg/mL and 516.45 mg/mL, respectively. These results, obtained from the DPPH radical scavenging assay, clearly demonstrate the superior antioxidant potential of the *G. lucidum* extract compared with the collagen peptides derived from *R. pulmo*. For comparative purposes, the results are illustrated in Figure 8C. Statistical analysis using one-way ANOVA, followed by *t*-test comparisons between test groups, revealed a significant difference only between JPC and the E2 (GL–JPC) biocomposite (*p* = 0.01 < 0.05), indicated by the symbol *.

#### 2.8.2. Reducing Power

**Figure 9:**
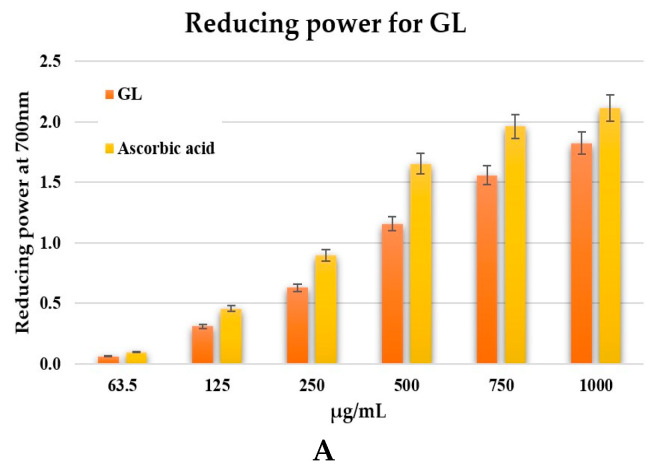

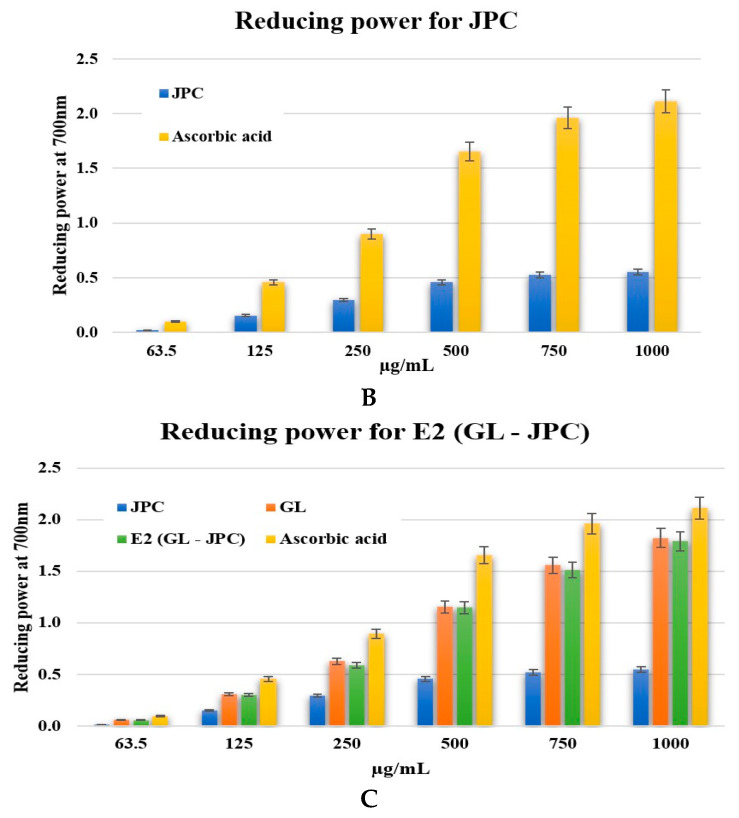
Reducing power test of *G. lucidum*–GL (**A**), *R. pulmo* collagen–JPC (**B**)**,** and newly formulated biocomposite GL-JPC (**C**) compared with ascorbic acid (standard antioxidant). Results represent the mean ± SD for *n* = 3.

The antioxidant capacity was also evaluated by the reducing power test, and the results are presented in Figure 9. The results for the reducing power exhibited by the ethanolic extract of *G. lucidum* are presented in Figure 9A, and Figure 9B presents the results for the reducing power of collagen extracts. Figure 9C compares the reducing power values of the individual components (*G. lucidum* and JPC) and the newly formulated GL–JPC biocomposite, using ascorbic acid as the reference standard. Statistical analysis performed using one-way ANOVA followed by t-tests revealed no statistically significant differences among the analyzed samples. These observations emphasize the relevance of developing new formulations, as the biocomposite demonstrates synergistic interactions between the bioactive compounds found in its individual components.

### 2.9. Biological Evaluation of the New GL-JPC Biocomposite

The antitumor activity of the *G. lucidum* (GL) extract and the biocomposites E1 (GL–JPC) and E2 (GL–JPC) was evaluated using oral squamous cell carcinoma (OSCC) cell lines, specifically SCC-9 and HSC-3. The experiments were conducted in accordance with ISO 10993-1 guidelines (Geneva, Switzerland, 2018) [59].

#### 2.9.1. Anticancer Activity on the SCC-9 Lines Cells of New Composite E1 and E2 with GL-JPC

**Figure 10:**
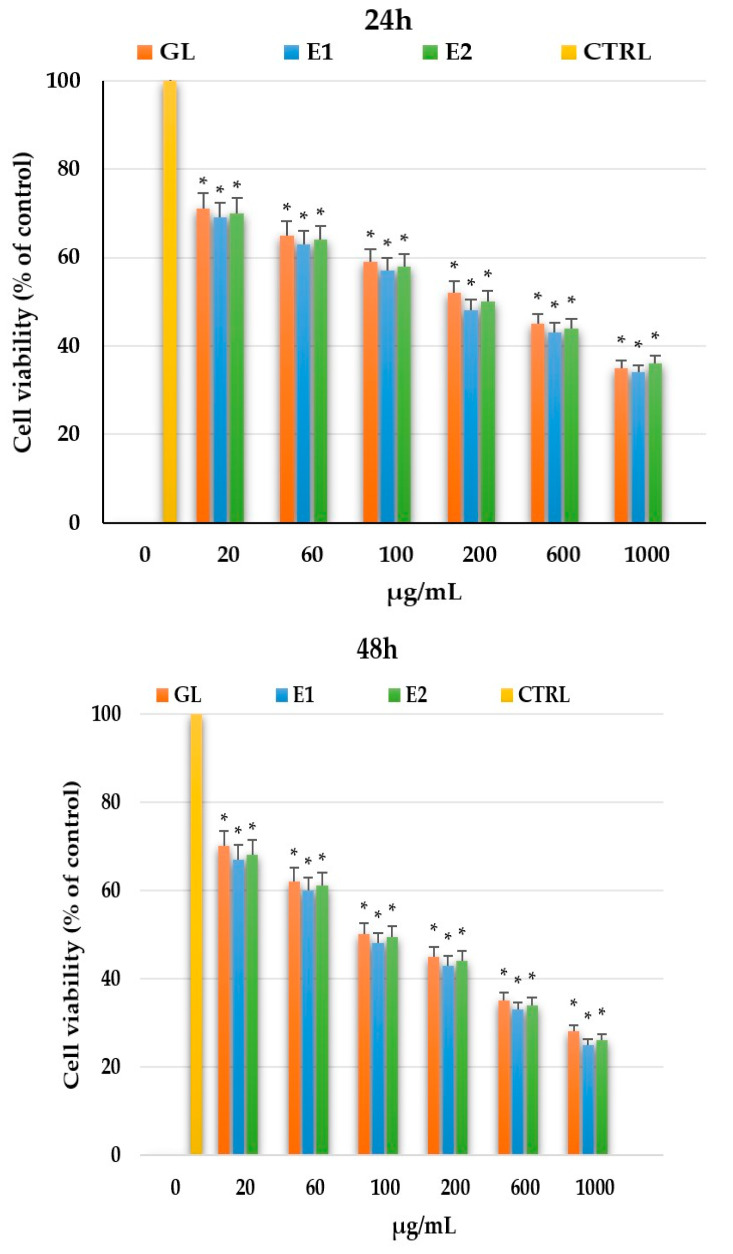

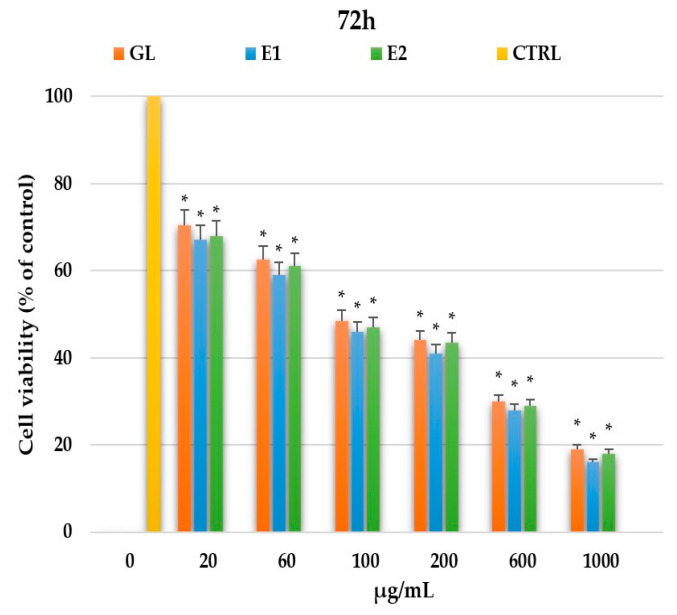
Cell viability in oral squamous cancer cell (OSCC) lines cells SCC-9, exposed to *G. lucidum* extract (GL), E1 (GL-JPC), and E2 (GL-JPC) extract in the 20–1000 µg/mL concentration range. Values are expressed as mean ± SD of 8 replicates (*n* = 8). * *p* < 0.05 for GL, E1 and E2 camparate with CTRL.

For the evaluation of anticancer activity against oral squamous cell carcinoma (OSCC), SCC-9 cell lines were used to assess cell viability (% of control) across a concentration range of 20–1000 µg/mL. SCC-9 cells, a human cell line derived from a metastatic lymph node, are commonly employed in OSCC research. Cytotoxicity was measured using the MTT assay. Cell viability results for the *G. lucidum* extract (GL) and the two composite formulations, E1 (GL–JPC) and E2 (GL–JPC), prepared with different component ratios, and the positive control substance, cisplatin, are presented in Figure 10. The control group consisted of SCC-9 cells cultured in medium supplemented with 0.5% fetal bovine serum.

Figure 10 illustrates the antitumor effects of the tested samples, with the GL extract showing the strongest cytotoxic response. Both E1 and E2 formulations exhibited similar inhibitory effects on SCC-9 cell viability, with significant reductions observed at concentrations above 100 µg/mL. Cell viability progressively decreased over time, namely at 24 h (from 69 to 71% to 32–35%), at 48 h (from 66 to 68% to 26–28%), and at 72 h (from 64 to 66% to 18–19%). These results demonstrate the potent, time and dose-dependent cytotoxic effects of both the GL extract and the GL–JPC biocomposites on OSCC cells.

Cell viability decreased progressively over time, namely at 24 h (from 69 to 71% to 32–35%), at 48 h (from 66 to 68% to 26–28%), and at 72 h (from 64 to 66% to 18–19%). Although the GL extract exhibited a more potent antitumor effect than the newly developed E1 and E2 biocomposites (GL–JPC), the antitumor activity of the composites remained substantial, showing only slightly lower efficacy compared with GL alone. Statistical analysis using ANOVA and *t*-tests indicated significant differences (*p* < 0.05), marked with an asterisk (*) for each sample (GL, E1, and E2) compared with the control (CTRL).

**Figure 11:**
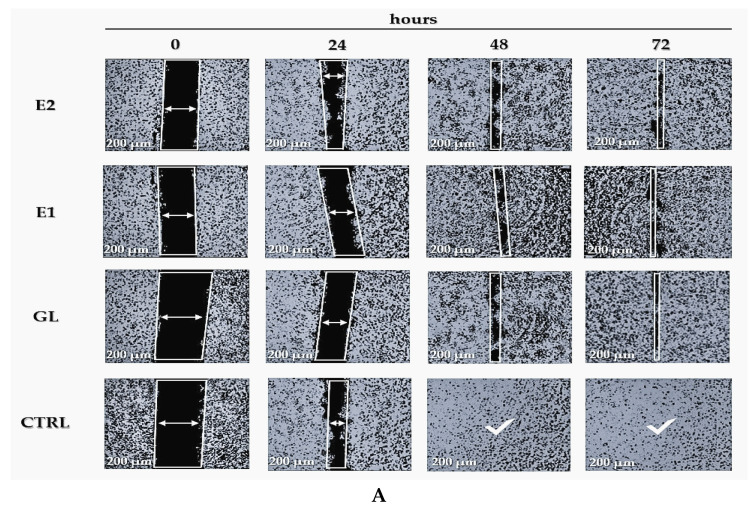

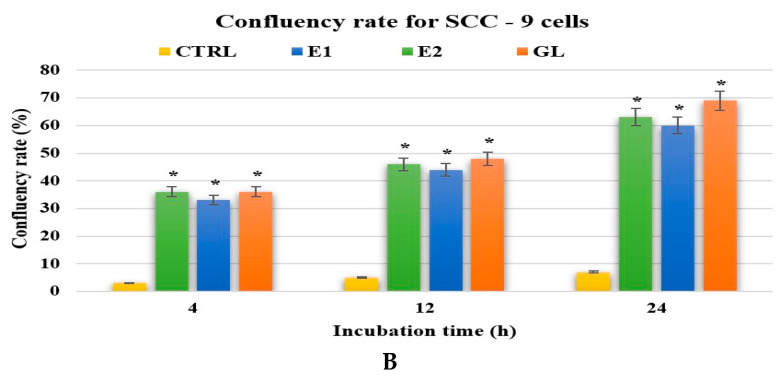
The results of micrographs of SCC-9 cells (treated and control). (**A**) Microscopic images from the scratch migration assay showing wound closure in SCC-9 cells, treated and untreated (CTRL), at 24, 48, and 72 h post-scratch. (**B**) Confluency rate of SCC-9 cells, expressed as the percentage of scratch closure relative to the initial wound area. The results are presented as mean ± SD for *n* = 8. The one-way ANOVA program was used for statistical analysis for *p* < 0.05, and the statistically significant differences for GL, E1, and E2 compared to the control group (CTRL) were indicated with the symbol (*).

Figure 11 presents the results of the migration assay performed on treated SCC-9 cells and untreated control cells, evaluated using the scratch test at 24 h, 48 h, and 72 h. The scratch assay, widely employed in wound-healing studies, assesses cell migration and layer regeneration at the wound margins through proliferative processes, as described by Pesterau et al. (2025) and Migone et al. (2022) [37,60]. Grada et al. (2017) further demonstrated the applicability of this method in assessing cell migration dynamics [61].

The migration capacity of SCC-9 cells treated with natural biocomponent extracts (samples GL, E1, and E2) was evaluated in comparison with untreated control cells (CTRL).

It was found that treatments with GL, E1, and E2 delayed the migration of cancer cells compared to the untreated control group. In the absence of treatment, CTRL cells were active for only 24 h. Cells treated with GL, E1, or E2 showed prolonged activity for up to 72 h in the affected area.

Figure 11B illustrates the confluency rate of SCC-9 cells in treated and untreated groups.

Statistical analysis using ANOVA and *t*-tests revealed significant differences (*p* < 0.05) in the confluency rates of GL-, E1-, and E2-treated samples compared to the CTRL group, as indicated by the asterisk (*).

In Figure 11A at 48 and 72 h, the CTRL samples showed no activity, while the GL and E1 and E2 formulations showed activity, indicating inhibitory effects on cancer cell movement.

#### 2.9.2. Anticancer Activity on the HSC3 Lines Cells of New Composites E1 and E2 with GL-JPC

**Figure 12:**
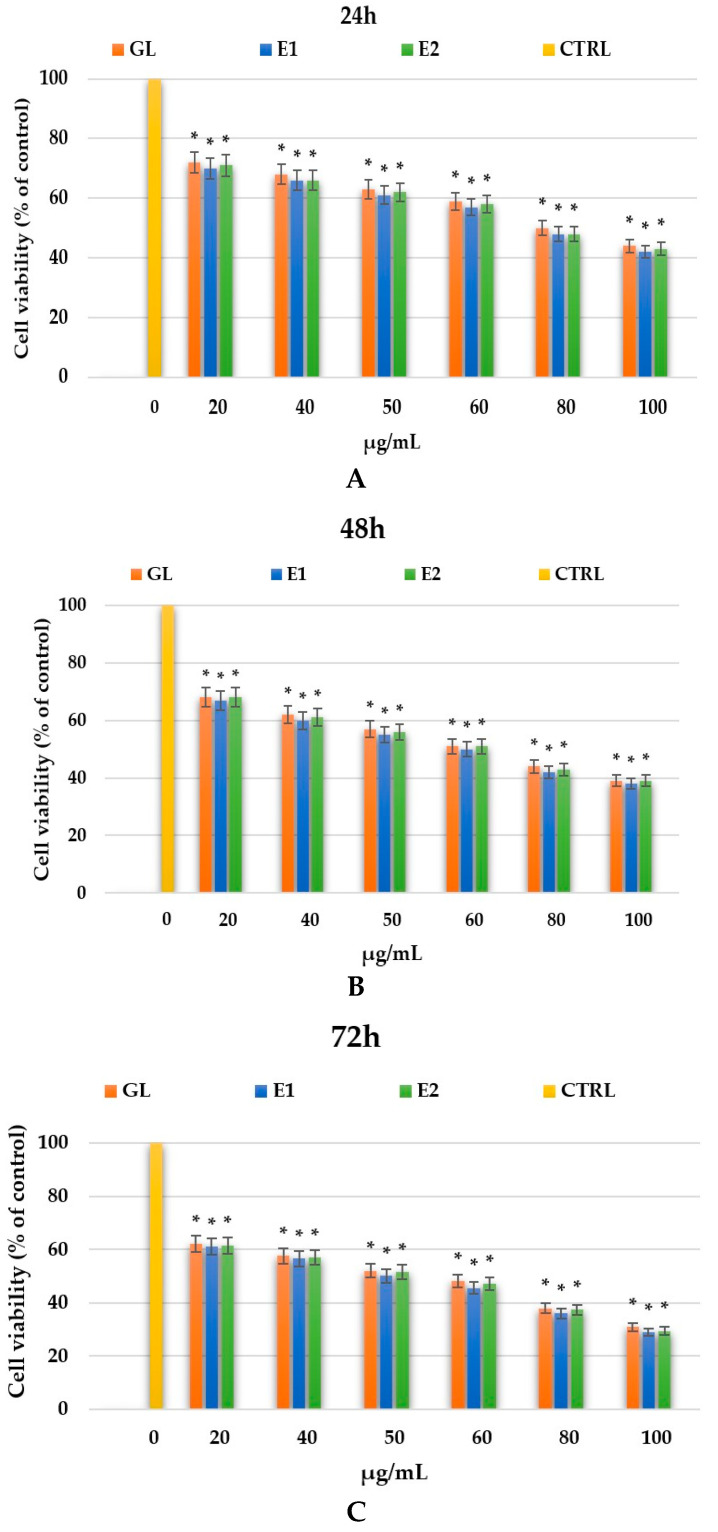
Cell viability (% of control) in oral squamous cancer cell (OSCC) lines cells HSC3 I, in a 20–100 µg/ mL concentration range. (**A**) Cell viability at 24 h for GL, E1, and E2; (**B**) cell viability at 48 h for GL, E1 and E2 GL, E1 and E2; (**C**) cell viability at 72 h for GL, E1, and E2. * for *p* < 0.05 for GL, E1 and E2 compared with CTRL. Data are presented as mean ± SD (*n* = 8).

The HSC-3 cell line represents an oral squamous cell carcinoma (OSCC) model derived from a human tongue tumor. A preliminary cytotoxicity evaluation of the GL extract and the biocomposites E1 (GL–JPC) and E2 (GL–JPC), and the positive control substance, cisplatin, was conducted on HSC-3 cells. Figure 12 illustrates cell viability at 24, 48, and 72 h for the GL, E1, and E2 test groups. Cell viability was reported as a percentage compared to the control and assessed for samples treated with the GL extract and with the E1 and E2 composites containing GL–JPC in variable ratios. For all tested samples, cell viability decreased progressively with both increasing concentration and longer exposure times.

**Figure 13:**
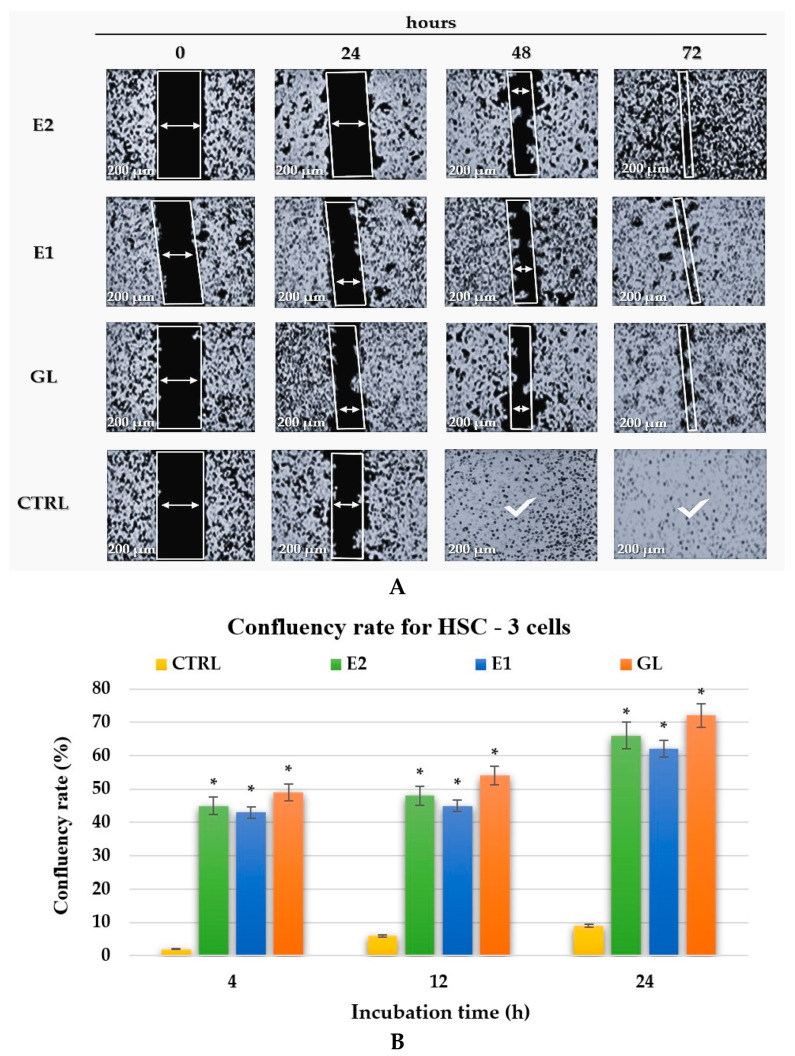
Migration assay of HSC-3 oral squamous cell carcinoma (OSCC) cells treated with *G. lucidum* extract (GL) and biocomposites E1 and E2 (GL–JPC) formulated with different component ratios. (**A**) Representative microscopic images from the scratch assay showing wound closure in treated and untreated (CTRL) HSC-3 cells at 24, 48, and 72 h post-scratch. (**B**) Confluency rate (%) of HSC-3 cells illustrating the delay in wound closure following treatment with GL, E1, and E2. Statistical analysis (ANOVA and *t*-test) revealed significant differences (* *p* < 0.01) for GL, E1, and E2 compared with CTRL. Data are presented as mean ± SD (*n* = 8).

The migration capacity of HSC-3 cells was further examined using the scratch assay for both treated (GL, E1, and E2) and untreated (CTRL) groups, as shown in Figure 13A Treatments with GL, E1, and E2 significantly slowed cancer cell migration compared with untreated controls during the first 24 h. In the absence of treatment, CTRL cells completely ceased activity within 48 h, while treated cells continued to fight cancer cells for up to 72 h. Similar results were reported by Camargo et al. (2022, 2023) [31,62], confirming the inhibitory effects of natural biocomponents on OSCC cell migration.

Figure 13B shows the confluency rate for treated and untreated HSC-3 cells. The GL, E1, and E2 treatments all resulted in significantly reduced confluency compared with the control (*p* < 0.01).

The CTRL samples acted for only 24 h, while the cells treated with GL, E1, or E2 showed slower migration and proliferation of cancer cells, acting for up to 72 h, as shown in Figure 13A.

## 3. Discussion

The most common type of oral cancer is oral squamous cell carcinoma (OSCC), as demonstrated by recent studies conducted by Badwelan et al. (2023) and Muralidharan et al. (2025) [63,64]. Conventional treatment strategies primarily include surgery, radiotherapy, and chemotherapy, while more recent approaches incorporate targeted therapy and immunotherapy. However, Muralidharan et al. (2025) emphasized that significant challenges persist, including tumor recurrence, metastasis, and treatment resistance [64]. Zhou et al. (2025) further noted that current studies often overlook the critical roles of cellular heterogeneity, drug resistance mechanisms, and the complex evolutionary trajectories of tumor development [65]. Tumor heterogeneity may occur from exposure to environmental factors—such as tobacco use, HPV infection, and dietary carcinogens—which contribute to cancer progression and the emergence of distinct cellular subpopulations with unique mutational profiles and therapeutic susceptibilities [65]. Additionally, certain food additives have been shown to exhibit cytotoxic effects, as reported by Busuricu et al. (2019) [66]. As a preventive measure, Warnakulasuriya et al. (2021) recommend that potentially malignant oral lesions (PMOLs), which have an increased risk of developing into cancer of the lips or oral cavity, be particularly carefully monitored and treated [3].

Several studies have focused on the prevention and management of oral complications associated with anticancer therapies, as well as on the monitoring and early detection of local recurrences, secondary tumors, or metastases in patients previously treated for oral cavity malignancies. Such work includes that of Cheng et al. (2021), Epstein et al. (2012, 2014), Ho et al. (2013), and Rethman et al. (2010) [11,67,68,69,70]. During chemotherapy or radiotherapy, multiple complications may arise that compromise the integrity of the mucosal barrier, thereby increasing the risk of systemic infections. Radiotherapy remains an essential therapeutic modality in cancer management; however, its adverse effects are considerable and often cumulative, affecting healthy tissues and leading to complications such as mucosal fibrosis, hyposalivation, and particularly osteoradionecrosis. Chang et al. (2017) and Reuss et al. (2023) reported that prophylactic interventions performed before radiotherapy can significantly influence the incidence of maxillary osteoradionecrosis (ORN) [71,72]. Moreover, Dunnack et al. (2021) demonstrated that alterations in the oral microbiome, which promote persistent dysbiosis, may facilitate the development of new or recurrent lesions [73].

Given these limitations of current therapeutic strategies, increasing attention has been directed toward alternative therapies, such as those based on natural biocomposites that can support tissue regeneration and help restore microbial balance in the oral cavity. These emerging therapies hold promise as adjuncts or complements to conventional cancer treatments, potentially improving both local healing and systemic outcomes.

In this context, innovative biomaterials, such as the bioactive composites developed in this study—based on collagen and biocomponents from *G. lucidum*—are an important idea to consider.

The use of collagen from marine sources for medical use has been emphasized by several authors, including Cadar et al. (2024), Bechir et al. (2014), and Sîrbu et al. (2010) [39,74,75]. Additionally, Cadar et al. (2023a) reported the anticancer effects and mechanisms of action of *G. lucidum* biocomposites [21]. The *G. lucidum* species has been extensively studied for its antitumor activity across various cancer models, as demonstrated by Camargo et al. (2022), Deng et al. (2021), and Jeitler et al. (2020) [31,76,77].

The chemical composition of *G. lucidum* can vary quantitatively depending on factors such as harvest season, growth stage, geographical origin, and substrate, as reported by Pascale et al. (2024a, 2024b) [78,79]. In our study, the *G. lucidum* extract was found to contain a high proportion of carbohydrates (42.80 ± 5.53%), followed by proteins (7.49 ± 0.56%), fibers (2.90 ± 0.01%), and lipids (2.83 ± 0.18%). In comparison, the protein extract obtained from the jellyfish *R. pulmo* mainly contained collagen (55.5 ± 0.6%).

The formulation of the new biocomposite based on *G. lucidum* ethanolic extract and jellyfish (*R*. *pulmo*) collagen extract develop a novel composition distinct from the individual components, resulting from the symbiotic interaction between the bioactive compounds of both species. This synergy involves the combination of polysaccharides and polyphenols from *G. lucidum* with the proteins and polyphenols present in *R. pulmo*, generating a material with enhanced biofunctional properties.

The polysaccharide profile of *G. lucidum* revealed the presence of maltose (51.01 ± 0.01 mg/g), fructose (14.55 ± 0.02 mg/g), glucose (12.91 ± 0.01 mg/g), sucrose (0.82 ± 0.01 mg/g), and xylose (0.41 ± 0.01 mg/g). Among these, maltose was identified as the predominant sugar, followed by glucose and fructose. Similar findings were reported by Li et al. (2020) and Liu et al. (2022), who confirmed the presence of glucose in *G. lucidum* extracts, while Sui et al. (2016) identified glucose, xylose, and fructose in *G. lucidum* polysaccharide fractions [80,81,82]. Zhong et al. (2024) and Li et al. (2018) further suggested that variations in the quantitative composition of *G. lucidum* polysaccharides may be attributed to differences in cultivation conditions, strain variability, and extraction techniques, including chromatographic separation methods [83,84].

Collagen peptides extracted from *R. pulmo* were analyzed by SDS-PAGE, confirming the presence of type I collagen. This finding is consistent with reports by Pesterau et al. (2025) and James et al. (2023) [37,57]. Other authors have also reported data on the fungus *R pulmo,* such as Addad et al. (2011), Paradiso et al. (2019), and De Domenico et al. (2019) [85,86,87]. The structure of collagen was verified using CDS and FT-IR spectroscopic techniques.

From the study of the amino acid composition, we found that the collagen from *R pulmo* we studied contained 16 amino acids, the most abundant of which was Gly. James et al. (2023) also presented similar results [57].

The analysis of the results regarding the protein content obtained for these two biomaterials shows that it is substantially higher in *R. pulmo* extracts (59.48 ± 1.72%) than in *G. lucidum* extracts (7.49 ± 0.56%), where it is relatively low. For *G. lucidum*, Peng et al. (2024) reported results for amino acid content, which confirm their presence in the fungus composition [88].

Cadar et al. (2023c) demonstrated that oral tissue regeneration and wound closure are largely favorized by collagen peptides extracted from *R. pulmo*, particularly glycine and hydroxyproline, which play essential roles in tissue repair processes [36]. Similarly, Ren et al. (2025) reported that amino acids present in *G. lucidum* contribute significantly to protein synthesis and exhibit antioxidant and hypoglycemic properties [89]. Moreover, the same study evidenced the presence of bioactive oligopeptides in *G. lucidum* with demonstrated anticancer effects through the induction of apoptosis in cancer cells [89]. The well-documented antitumor and antioxidant properties of *G. lucidum* have also been confirmed by Toson et al. (2025), Haleem et al. (2024), and Hsu et al. (2021) [22,30,32].

Comparing the two species, the total polyphenol content of *G. lucidum* was 35.80 mg GAE/g DW, whereas for *R. pulmo* it was 6.58 mg GAE/g WW. The results for *G. lucidum* are consistent with data reported by Rahman et al. (2020), Sheikh et al. (2015), and Wu et al. (2015) [46,90,91]. The TPC value for *R. pulmo* collagen extract was comparable to the findings of Leone et al. (2015) [51]. For *G. lucidum,* the TFC was similar to that reported by Gąsecka et al. (2020) [58]. These results indicate that *G. lucidum* contains a significantly higher concentration of phenolic compounds than *R. pulmo*, supporting its superior antioxidant potential. Recent investigations by Pascale et al. (2024a) have likewise emphasized *G. lucidum* as a plentiful source of secondary metabolites with potent biological activity [78].

In total, nine individual polyphenols were identified in the *G. lucidum* extract, while three were identified in the *R. pulmo* extract. Among the compounds found in *G. lucidum*, gallic acid was the most abundant phenolic acid, in agreement with the findings of Veljović et al. (2017) [92].

The results of the macroscopic and microscopic analyses were influenced both by the intrinsic characteristics of the individual ingredients and by the formulation parameters, specifically, the ratios between the *G. lucidum* extract and the collagen peptides extracted from the jellyfish *R. pulmo*. The final formulations demonstrated distinct organoleptic and structural characteristics. The color intensity of the GL–JPC biocomposites increased proportionally with the concentration of fungal extract.

Microscopic analysis revealed a progressive interaction between the plant and marine extracts at the initial stage of mixing (time 0), which evolved toward a uniform and well-integrated composite structure. Rheological characterization of both formulations—E1 (GL–JPC) and E2 (GL–JPC)—showed pH values of 6.8 and 6.7, respectively. The rheological profiles confirmed their suitability for pharmaceutical applications. It was observed that the formulated hydrogels retained their structural and rheological properties over time, exhibiting flow behavior according to the Ostwald–de Waele rheological model (power law) with the presentation of hysteresis loops. These findings are in accordance with previously reported studies describing similar rheological behavior in hydrogels formulated with plant-derived materials and marine collagen, as reported by Cherim et al. (2019) and Cadar et al. (2019b) [35,93]. Comparable results for viscous formulations based on marine collagen were also documented by Cherim et al. (2019), Pesterau et al. (2025), and Sirbu et al. (2010) [35,37,75].

The antimicrobial activity of *G. lucidum* (GL), jellyfish collagen peptides (JPC), and the biocomposite E2 (GL–JPC) was evaluated using the agar diffusion method against common oral pathogens. The results showed that against S. mutans, an inhibition zone of 18 ± 0.5 mm was observed. For the biocomposite E2 (GL–JPC), it was slightly smaller than that obtained for GL (20 ± 0.5 mm) but significantly higher than JPC alone (10 ± 0.2 mm). The second most sensitive strain was *S. aureus*, with inhibition zones of 17 ± 0.2 mm for E2 (GL–JPC), 19 ± 0.5 mm for GL, and 11 ± 0.3 mm for JPC. For *C. albicans*, the inhibition zones were 17 ± 0.2 mm for E2 (GL–JPC), 18 ± 0.5 mm for GL, and 10 ± 0.5 mm for JPC. The antimicrobial activity of *G. lucidum* extracts against various bacterial strains has also been described by Karunarathna et al. (2025), Ahmad et al. (2024), Hoque et al. (2015), Avci et al. (2014), and Kamra et al. (2012) [94,95,96,97,98].

The antimicrobial activity of the three samples revealed differences between the individual compounds and the formulated biocomposite, E2 (GL-JPC). The biocomposite had an intermediate antimicrobial response, while GL responded best to the inhibition of pathogenic strains and JPC showed modest antimicrobial activity, indicating an additive effect of the two compounds rather than a strong synergistic effect.

Simões et al. (2018) emphasized that for formulations intended for application in the oral cavity, particularly on wounds or mucosal surfaces, the evaluation of antimicrobial properties is essential [99]. Pesterau et al. (2025) noted that differences in bacterial cell membrane structure can influence the antimicrobial response of various bacterial strains [37]. Similarly, Erbiai et al. (2023) reported that *S. aureus* exhibits higher sensitivity to *G. lucidum* extracts compared to *C. albicans* [100]. Mojarad et al. (2025) demonstrated a significant antimicrobial effect of the methanolic extract of *G. lucidum* against *S. mutans*, supporting its potential use in oral care applications [101]. Comparable findings were also reported by Pesterau et al. (2025), Kamra et al. (2012), Parameswari et al. (2024), and Sridhar et al. (2011) [37,98,102,103].

The different mechanisms of action of the biocomposite can be observed through the results obtained following the inhibition of pathogenic strains, which is attributed to the structural and biochemical differences in the bacterial cell envelope. Thus, we mention that for Gram-positive bacteria that have a thick peptidoglycan layer, E2 and GL had remarkable antimicrobial activity, and this is justified by the increased permeability of the cell membrane to phenolic compounds and peptides that can alter its integrity and interfere with protein synthesis. Gram-negative bacteria may have a relatively lower susceptibility due to their lipopolysaccharide-rich membrane [37].

The effectiveness of the E2 (GL-JPC) biocomposite on pathogenic strains may be due to compounds with antimicrobial activity, such as polyphenolic compounds and sterols, organic acids, or fatty acids, which are found in the composition of the fungus [100]. The effectiveness of JPC on some bacterial strains may be due to the presence of collagen-derived peptides that can exert a weak interactive effect on the membrane, especially against Gram-positive bacteria [37]. Recent studies have shown that the antimicrobial efficacy of the *G. lucidum* mushroom depends heavily on the accessibility of the biocomponents extracted by different extraction methods and the extraction solvent, as well as the formulation of *G. lucidum*-based products [98]. Mojarad et al. (2024) showed that a formulation based on *G. lucidum* and nisin has an additive but not synergistic effect on bacterial growth inhibition, despite the fact that activities in other biological criteria are improved [101]. The results presented in this study provide initial evidence of the innovative biocomposite’s antimicrobial activity, but further investigations on a wider range of bacterial strains are needed to provide a complete characterization of its antimicrobial potential.

The study for antioxidant capacity demonstrated that the new GL–JPC composite exhibited an antioxidant capacity comparable to that of the GL extract and significantly superior to that of the JPC collagen peptide extract, as shown in Figure 8C and Figure 9C. Sirbu et al. (2019b) and Cadar et al. (2019c) reported similar results for marine-derived bioactive materials, highlighting the essential role of polyphenols and polysaccharides in determining antioxidant potential [26,104]. The antioxidant capacity of *G. lucidum* extracts has been extensively studied by several authors, including Fan et al. (2012), Ma et al. (2013), and Kang et al. (2019) [105,106,107]. Furthermore, Cör et al. (2018) demonstrated that the antioxidant, antimicrobial, and antitumor effects of *G. lucidum* are predominantly mediated by terpenoids and polysaccharides [108]. The excess free radicals can damage nucleic acid bases and amino acid residues in proteins, as demonstrated by Cadar et al. (2023a) [21]. In this context, Hsieh et al. (2011) suggested that bioactive compounds derived from *G. lucidum* with antioxidant properties may have chemopreventive actions [109].

Smina et al. (2011a) investigated the antioxidant activity of *G. lucidum* extracts and demonstrated their ability to neutralize free radicals within cancer cells [110]. In a subsequent study, Smina et al. (2011b) reported the protective role against radiation due to triterpenes in *G. lucidum* [111]. Similarly, Lu et al. (2001, 2003) highlighted the important role of polysaccharides from *G. lucidum* in combating ROS [112,113].

Other researchers, such as Lee et al., have shown that hydroxyl radicals and superoxide anions can be inactivated by a compound with an amino-polysaccharide structure found in *G. lucidum* [114]. Furthermore, Xiaoping et al. and Zhao et al. reported that *G. lucidum* bioactive compounds improved glutathione peroxidase activity and reduced malondialdehyde levels in rats with carcinoma and in mice subjected to γ-radiation, supporting their systemic antioxidant and radioprotective properties [115,116].

The in vitro biological evaluation focused on assessing the cytotoxic effects of the tested samples: GL, E1 (GL-JPC), and E2 (GL-JPC) on oral squamous cell carcinoma (OSCC) cell lines, specifically SCC-9 and HSC3, at various concentration ranges. The results obtained for SCC-9 cells shot at 20 µg/mL showed that the cell viability decreased from 69 to 70% after 24 h to 67–68% after 72 h, while at 1000 µg/mL it dropped from 34 to 35% at 24 h to 16–18% at 72 h. The confluence rate values for SCC-9 cells were between 53% (E2) and 60% (GL) after 24 h, increasing to 79% (E2)–85% (GL) after 48 h. For HSC3 cells, the experiments were conducted in the 20–100 µg/mL concentration range. Cell viability decreased progressively over the 24–72 h period, from 72 to 70% at 24 h to 62–61% at 72 h for the 20 µg/mL concentration. At 100 µg/mL, viability dropped from 42% (24 h) to 29% (72 h) for extracts containing bioactive compounds. The confluence rate at 24 h ranged between 66% (E2) and 72% (GL).

According to the specialized literature, collagen peptides derived from jellyfish also possess antitumor activity, although their effects are weaker compared to the bioactive compounds from *G. lucidum*, as demonstrated by Prommasith et al. (2024) and Khalil et al. (2022) in studies using collagen hydrolysates on different tumor cell lines [117,118].

Other authors have also shown that the biocomponents in *G. lucidum* have antitumor activity. The cytotoxic effect of the ethanolic extract of *G. lucidum* was studied by Dwiandhono et al. (2020) on KB CCL-17 oral tumor cell lines, with a marked reduction in cell viability at higher concentrations (1000 µg/mL) and an IC_50_ value of 8.49 µg/mL [119]. Similarly, Camargo et al. (2022) investigated the antitumor potential of polysaccharide fractions extracted from *G. lucidum* on SCC-9 tongue carcinoma cells and demonstrated a concentration-dependent cytotoxicity in the range of 0.01–15 mg/mL, with cell viability dropping below 50% at 15 mg/mL [31]. In a subsequent study, Camargo et al. (2023) evaluated *G. lucidum* polysaccharides (5 mg/mL and 10 mg/mL) in combination with fluorouracil and observed a significant reduction in cell viability at 10% biocompound concentration [62]. Comparable results were reported by You et al. (2012) for *G. lucidum* extracts tested on HSC3 oral cancer cells, where inhibition of proliferation and migration was observed [120].

Xu et al. (2023) characterized the HSC3 cell line as originating from human tongue carcinoma with lymph node metastasis, emphasizing its aggressiveness and suitability as a model for studying oral cancer progression, cellular motility, and the evaluation of novel anticancer agents [121]. Kim et al. (2024) conducted in vivo studies on mice with periodontitis and found that *G. lucidum* spore oil significantly reduced periodontal inflammation and minimized alveolar bone loss, associated with decreased MMP-1 and IL-8 levels [29]. Haleem et al. (2024) demonstrated in vitro antioxidant and antitumor activity of three different *G. lucidum* solvent extracts (cold distilled water, acidified water with 0.1 M HCl, and 70% ethanol) against human oral squamous cell carcinoma (HOSCC) and human skin squamous cell carcinoma (HSSCC) cell lines. The most pronounced cytotoxicity was observed with the acidified water extract, particularly against HOSCC cells, and the effect was both concentration- and time-dependent [30]. Zeng et al. (2020) further confirmed that *G. lucidum* polysaccharides exhibit significant antitumor activity against oral squamous cell carcinoma (OSCC) [33].

In this study, the antitumor activity of the three tested samples was observed on the SCC9 and HSC3 tumor cell lines. According to the specialized literature, the bioactive constituents involved in the antitumor effect are mainly polyphenolic compounds, triterpenes, and polysaccharides found in the composition of the mushroom [119]. Dwiandhono et al. (2020) determined the antitumor activity of the ethanolic extract of *G. lucidum* compared to the cisplatin standard, on oral cancer cells KB CLL-17, and the results showed comparable effects of the extract with those of the standard and suggested a direct inhibition of the metabolic activity of tumor cells. This effect may be associated with triterpene compounds, thought to be involved in inducing cell cycle arrest and apoptosis through mitochondrial pathways, including p53 activation, modulation of the BAX/BCL-2 balance, cytochrome-c release, and caspase cascade activation [119]. Similarly, the antitumor activity may also be attributed to the polysaccharides in the mushroom composition, demonstrated by studies on OSCC tumor lines (SCC9) that determined an antitumor mechanism related to the functional reprogramming of tumor behavior [31].

More specifically, polysaccharides extracted from the fungal species suppress proliferation, migration, and reduce markers of tumor stem cells, epithelial–mesenchymal transition, and ABC transport, and they also induce cellular senescence without triggering apoptosis [31]. These findings indicate that *G. lucidum* possesses remarkable antitumor effects on tumor cells taken from the oral cavity through multiple mechanisms, dependent on the extracted compounds, and, at the same time, support the use of *G. lucidum* extracts as a source of bioactive compounds with antitumor potential in oral squamous cell carcinoma models. This preliminary in vitro study demonstrates that the new biocomposite exhibits measurable, dose-dependent cytotoxic activity.

This study has several limitations that should be acknowledged. First, the biological evaluations were restricted to in vitro assays using OSCC cell lines, without comparison to healthy oral cells, which limits conclusions regarding therapeutic selectivity. Second, the antimicrobial synergy observed against *Staphylococcus aureus* was identified in a limited experimental context and requires further validation and mechanistic exploration. Third, no in vivo studies were conducted to assess biocompatibility, biodistribution, or long-term safety. Despite these limitations, the results provide a structured basis for future investigations aimed at optimizing the formulation, elucidating mechanisms of action, and evaluating clinical relevance.

## 4. Materials and Methods

The fungal species *G. lucidum* and the jellyfish species *R. pulmo* were the natural resources used to obtain a novel biocomposite to be used as a potential alternative therapeutic agent for oral squamous cell carcinoma (OSCC). Both species were collected from distinct Romanian ecosystems. *G. lucidum* fruiting bodies were harvested from the mountainous region near Bistrița-Năsăud, Romania. The *R. pulmo* jellyfish originate from the waters of the Black Sea, more precisely from the coast of Constanța City.

### 4.1. Chemical Reagents

Specific reagents were employed for each experimental method and processing step. All reagents and controls were of analytical grade. The reagents for the SDS-PAGE analysis were obtained from Bio-Rad Laboratories (Hercules, CA, USA). All other reagents were purchased from Sigma-Aldrich (Darmstadt, Germany) and Merck KGaA (Darmstadt, Germany). Standards used for the determination of amino acids and of polyphenols were also obtained from Sigma-Aldrich (Saint Louis, MO, USA). All materials used in the antimicrobial study were donated by the Veterinary and Food Safety Directorate, Constanța, Romania. The line SCC-9 cells were obtained from ATCC (New York, NY, USA), while HSC3 cells were purchased from Merck KGaA (Darmstadt, Hessen, Germany). For cell activation, SCC-9 and HSC3 cells were cultured in Dulbecco’s Modified Eagle Medium (DMEM; Thermo Fisher Scientific, Waltham, MA, USA) supplemented with 10% fetal bovine serum (FBS; Thermo Fisher Scientific, Waltham, MA, USA) and 1% penicillin–streptomycin (Thermo Fisher Scientific, Waltham, MA, USA).

### 4.2. Preparation of Extracts

#### 4.2.1. *G. lucidum* Extracts

The protocol described by Pascale et al. (2024a, 2024b) and Cadar et al. (2019b) was followed with minor modifications to obtain the *G. lucidum* extracts [78,79,93]. For aqueous extraction, the hot water extraction (HWE) method was applied. The mushroom fruiting bodies were thoroughly washed, dried, minced, and subjected to extraction with double-distilled water under continuous stirring for 24 h. The resulting mixture was filtered to obtain the *G. lucidum* aqueous extract.

For alcoholic extraction, a hot extraction procedure was performed using a Soxhlet apparatus (Fisher Scientific, Leicestershire, UK) with 70% ethanol as the solvent. The resulting solution was collected as the *G. lucidum* ethanolic extract (GL). A 10% (*w*/*v*) solution of *G. lucidum* in 70% ethanol was prepared and stored in tightly sealed amber containers at 4 °C until further analysis.

#### 4.2.2. *R. pulmo* Collagen Extract

Collagen extracts were obtained from *R. pulmo* specimens collected from the Black Sea. The samples were first subjected to pretreatment steps consisting of cleaning with NaCl solution, washing with ultrapure water, and demineralization with 0.5 M EDTA-Na solution. For collagen extraction, the protocol described by Cadar et al. (2024) was used, employing the PSC method with 10% (*v*/*v*) pepsin at 4 °C [39]. The reagents used for precipitation were 0.5 M acetic acid; for reprecipitation, 0.5 M NaCl solution; as well as 10% pepsin solution and ultrapure water. The precipitates were centrifuged for 1 h at 20,000 rpm. A 0.05 M Tris buffer (pH 7.0) was used.

The extraction and subsequent preparation of collagen peptides were performed following the methodologies described by Felician et al. (2019) [122]. Equation (1) was used to calculate collagen yield:

(1) $$Collagen\;yield\;\left(wet\right)\%=\frac{weight\;of\;extracted\;collagen\;(g)}{weight\;of\;wet\;jellyfish\;(g)}\times 100$$

Collagen was used either in the form of a hydrogel or lyophilized and stored as a powder for later use. Collagen peptides were obtained using a slightly modified procedure by Felician et al. (2019) [122]. Moreover, 1 g of collagen (PSC) dissolved in 200 mL of water was incubated at 37 °C in a water bath. Enzymatic hydrolysis was performed using collagenase type II at a 5% (*w*/*w*) enzyme-to-substrate ratio for 5 h. After 10 min of heating at 95 °C, the enzymatic reaction was stopped. The cooled mixture was centrifuged for 30 min at 3000 rpm. Collagen peptides (JPCs) from the supernatant were collected and stored at 4 °C until further use.

### 4.3. Formulation of the New Biocomposite Based on G. lucidum and R. pulmo

The formulation of the new biocomposites was carried out following the methods described by Sîrbu et al. (2019b) and Pesterau et al. (2025), with appropriate adaptations for mixing the plant extract with the marine collagen extract to obtain pharmaceutical formulations suitable for oral mucosal applications [26,37]. Two formulations were prepared: biocomposite **E1 (GL–JPC)**, consisting of 3/5 (*v*/*v*) parts 10% ethanolic *G. lucidum* extract and 2/5 (*v*/*v*) parts JPC collagenic hydrogel, and biocomposite **E2 (GL–JPC)**, consisting of 4/5 (*v*/*v*) parts 10% ethanolic *G. lucidum* extract and 1/5 (*v*/*v*) parts JPC collagenic hydrogel.

To obtain the biocomposites, the lyophilized collagen peptides were first brought into aqueous solution at a pH = 9. The peptide solutions were then combined with the ethanolic *G. lucidum* extracts according to the specified ratios. The mixtures were homogenized by stirring for 30 min at 50 °C and then centrifuged for 15 min at 10,000 rpm. The resulting biocomposites (E1 and E2) were prepared in hydrogel form and stored at 4 °C until further analyses.

### 4.4. Determination of the Proximal Composition and Nutritional Values of the Studied Species

The moisture content (%) and ash content (%) were obtained by applying standardized AOAC procedures [123]. To obtain the moisture content, the test sample was dried to constant weight. The ash content was obtained by incineration at 500 °C. The results were reported as percentages of the initial dry weight.

#### 4.4.1. Biochemical Composition of Fungus *G. lucidum*

The biochemical composition of *G. lucidum* (GL) was determined in terms of carbohydrate, protein, lipid, and fiber content following the methods described by Peng et al. (2024), Roy et al. (2015), and Singh et al. (2020), adapted to actual laboratory conditions [88,124,125]. The analytical approaches were consistent with those previously reported by Cadar et al. (2019d) [126]. Carbohydrate content was obtained using the procedure of Albalasmeh et al. (2013) [127]. Mix 1 mL of aqueous carbohydrate solution with 1 mL of 5% (*w*/*w*) phenol and 2.5 mL of concentrated sulfuric acid, and read the absorbance at 490 nm for the new yellow-orange complex. Reading is performed with a VWR UV-6300 PC double-beam spectrophotometer (Leuven, Belgium). According to Albalasmeh et al. (2013), a linear correlation exists between the carbohydrate concentration and the absorbance measured at 490 nm [127].

Equation (2) is the glucose standard calibration curve

y = 0.1009x − 0.0024(2)

where y is the carbohydrate concentration; x is the absorbance, and R^2^ = 0.992 is the correlation coefficient of curve.

The protein content was obtained with the Lowry method [128]. The BSA calibration standard was obtained from PAN Biotech GmbH (Aidenbach, Germany). Absorbance readings were recorded using a VWR UV-6300 PC double-beam spectrophotometer at wavelengths of λ = 750 nm and λ = 500 nm, depending on the protein concentration. The protein content was calculated using the corresponding standard curve, and the results were expressed as a percentage of the dry weight of the mushroom. This method was also applied by Rahman et al. (2020) and Roy et al. (2015) for the determination of crude protein in *G. lucidum* [46,124].

Lipid content was determined by Soxhlet extraction using petroleum ether as solvent for 5 h, following the procedure described by Roy et al. (2015) with gravimetrically method [124]. The lipid content was calculated as a percentage of the dry weight of the mushroom.

Fiber content was obtained by the method of Peng et al. (2024), which requires the removal of fats and soluble components using an acid–alkaline digestion mixture. [88]. The resulting residue was filtered, dried, and weighed. This method was also applied by Singh et al. (2020) [125]. The fiber content was expressed as a percentage of the dry weight of the mushroom.

#### 4.4.2. Biochemical Composition of Jellyfish *R. pulmo*

To determine the biochemical composition of the jellyfish species *R. pulmo*, the methodologies described by Leone et al. (2015) were applied [51]. And the methods of other researchers, such as D’Ambra et al. (2022) and Migone et al. (2022), were adapted to the conditions used in the laboratory [53,60]. To obtain the total protein content, the method of De Domenico et al. (2019) was used, which was based on the method of Bradford (1976) [87,129].

Protein extraction, hydrolysis, and fractionation were carried out prior to quantification. Absorbance readings were performed using an Infinite M200 Quad4 Monochromator Detection System (Tecan, Männedorf, Switzerland). The calibration standard was bovine serum albumin (BSA). Results were presented as mean ± SD based on three independent determinations. It is noteworthy that for *R. pulmo*, the proteins represent the most abundant class of biochemical compounds.

For lipids, the gravimetric method described by Percy et al. (1981) was used, applied to 100 mg of sample in a Soxhlet apparatus [130]. The resulting extract was washed with 1% NaCl solution, and the solvent residue was evaporated. The lipid fraction obtained was weighed. The results were reported as a percentage of the sample mass based on the average of three independent determinations. The method of Zhang et al. (2014) was used to obtain carbohydrate content [131]. Protein removal from jellyfish extracts was performed using papain and Savag reagent, followed by ethanol precipitation of polysaccharides at concentrations of 60%, 70%, and 80% (*v*/*v*). Polysaccharide extraction from *R. pulmo* tissue was carried out at 100 °C for 4 h. The variations in polysaccharide fractions may occur due to extraction conditions [60]. The final carbohydrate content was expressed as a percentage (mean ± SD) based on three independent measurements.

### 4.5. Identification of Monosaccharide Types of the Polysaccharide Content from G. lucidum

The determination of monosaccharides types of polysaccharides in the composition of the fungal species *G. lucidum* was performed using the HPLC-DAD method, adapted from the method of Li et al. (2020) [80]. For the analyses, an Agilent 1200 HPLC system (Agilent Technologies, Santa Clara, CA, USA) was used. The HPLC worked with a ChemStation (Chem 32) data integration software system (Agilent). The retention times (RT, ±SD) of the standard monosaccharides used for quantification were as follows: xylose–0.0967 ± 0.025 min, fructose–0.2131 ± 0.015 min, glucose–0.5278 ± 0.020 min, sucrose–0.5277 ± 0.015 min, and maltose–0.6488 ± 0.010 min. Polysaccharide identification in *G. lucidum* was obtained by comparing the retention times of the standards with those determined for the analyzed samples under identical HPLC-DAD operating conditions.

Results were expressed as mean ± standard deviation (SD) in mg/g dry weight (DW). Representative chromatograms of both the standard compounds and the identified monosaccharides in *G. lucidum* extracts are presented to illustrate peak correspondence and separation quality.

### 4.6. Jellyfish R. pulmo General Characterization

#### 4.6.1. SDS-PAGE Analysis

Jellyfish collagen was analyzed by SDS-PAGE. The protocol used is that described by Pesterau et al. (2025) [37]. This working technique, with some modifications, is also described by James et al. (2023) [57]. Other researchers, such as De Domenico et al. (2019), have also reported studies on the SDS-PAGE technique applied to jellyfish collagen [87]. For collagen from various jellyfish, Addad et al. (2011) reported studies performed by SDS-PAGE analyses [85]. Phosphate buffer was used to work at pH 7.4. A quantity of 10 g of the mixture with phosphate-buffered solution was centrifuged for 5 min and then subjected to dialysis. The extraction process was followed by heat treatment at 95 °C to denature the proteins. Laemmli reagent was used to prepare the samples prior to loading. Electrophoresis was performed for 30 min at 85 V and then 95 V until completion. The equipment used for electrophoresis was a calibrated GD-800 densitometer (Bio-Rad Laboratories, Hercules, CA, USA). Quality One software (version 4.6.3, Bio-Rad) was used for data processing. The resulting protein band profiles of the *R. pulmo* collagen extract are presented in Figure 3A.

#### 4.6.2. Circular Dichroism Spectral Analysis

Circular dichroism (CD) spectroscopy applied to collagen from *R. pulmo* allowed obtaining data on the secondary and tertiary structures of proteins from collagen extracted from *R. pulmo*. The CD spectroscopy method used was that reported by Pesterau et al. (2025) [37]. Measurements acquired with a Jasco J-810 UV-DC spectrophotometer (Jasco Corporation, Ishikawamachi, Tokyo, Japan) using quartz cuvettes (path length 0.02 cm). Spectra were recorded over the 250–195 nm wavelength range, at 25 °C.

The UV-CD spectrum of collagen showed a minimum at 198 nm and a maximum at 214.5 nm, consistent with a preserved secondary structure. CD analysis provides information on the protein’s secondary structural elements, confirming the integrity of the collagen triple helix. The results are presented graphically.

#### 4.6.3. FT-IR Spectroscopy Analysis of Collagen

The data obtained through FT-IR Spectroscopy allows confirmation of the molecular structure of collagen by identifying characteristic functional groups. To obtain the spectra, a Jasco 4200 FT-IR spectrometer (produced by Jasco Corporation, Ishikawamachi, Tokyo, Japan) was used. The equipment was equipped with Spectra Manager II software. Moreover, 0.5 M acetic acid was used as a solvent, and potassium bromide was used to obtain the analysis tablets. Spectra were recorded over the 700–4000 cm^−1^ range, with an extended instrument range of 350–7800 cm^−1^. The results are presented graphically and show information on the characteristic amide bands and other functional groups of collagen [37].

### 4.7. Determination of the Amino Acid Composition from R. pulmo

The method applied for determining the amino acid content of *R. pulmo* collagen was the one applied by Pesterau et al. (2025) [37]. Eid et al. (2022) also determined amino acid compositions by chromatographic methods [132]. The amino acid analysis methods followed the AccQ-Tag protocol (Waters, Milford, MA, USA). The chromatographic method was applied using HPLC equipment (type 626 LC System, Waters, Milford, MA, USA) [133]. This HPLC chromatograph was equipped with a 474 scanning, fluorescence detector, a 717 Plus autosampler, and Millennium Chromatography Manager software. Chromatographic separation was performed with a binary elution system on a C18 column. Operating conditions: column temperature of 37 °C; a flow rate of 1 mL/min; fluorescence detection at λ_ex = 250 nm and λ_em = 395 nm. Amino acid content was reported as residues per 1000 residues. Comparable methods have been applied to collagen peptides from other jellyfish species, such as *Chrysaora* sp. (Barzideh et al., 2014) [133].

### 4.8. Determination of the Polyphenolic Content

#### 4.8.1. Determination of the Total Phenolic Content (TPC)

TPC was obtained using the method of Sidhu et al. (2019), which is an adaptation of the Folin–Ciocalteu colorimetric method originally described by Singleton et al. [134,135]. The method was further adapted for *G. lucidum* by Peng et al. (2024) [88].

A quantity of 1 g of the sample was dissolved in 30 mL of anhydrous ethyl alcohol. This solution was sonicated under fixed conditions (25 min and 240W). It was transferred to a volumetric flask and adjusted to 50 mL. The next step consisted of mixing 1 mL of the filtered solution with 8 mL of double-distilled water and 1 mL of Folin–Ciocalteu reagent.

After 6 min at room temperature, 5 mL of 5% sodium bicarbonate solution was added. The solution was incubated for 90 min in the dark at room temperature. At 750 nm, the absorbance was read with an A. Kruss UV-VIS 6500 spectrophotometer. (A. Krüss Optronic GmbH, Hamburg, Germany).

TPC was calculated using a gallic acid standard curve with Equation (3)

(3) $$y=0.0018x-0.0578;\;R^{2}=0.9999$$

The results for TPC of *G. lucidum* were reported as µg GAE/g DW. All measurements were performed in triplicate. The TPC of *R. pulmo* (JPC) was also obtained using the Folin–Ciocalteu method. For the jellyfish collagen extracts, the same procedure was applied, with the exception that the calibration curve used for quantification corresponded to Equation (4):

(4) $$y=0.0067x-0.029;\;R^{2}=0.9998$$

Results were reported as µg GAE/g DW of JPC and represent the average of three successive determinations. Results were consistent with data reported by Leone et al. (2019) [51]. There were also Stabili et al. (2021) who reported data referring to TPC from jellyfish [136].

#### 4.8.2. Determination of the Total Flavonoid Content (TFC) from *G. lucidum*

To obtain the TFC values, we used the colorimetric method initially proposed by Chang et al. (2002), which was later adapted for the extract of *G. lucidum* by Masjedi et al. (2022) [137,138]. From the hydroalcoholic extract of *G. lucidum*, 0.5 mL of extract was mixed with 0.1 mL of 10% AlCl_3_ solution, 1.5 mL of methyl alcohol, and 0.1 mL of 1M CH_3_COOK solution. After diluting with 2.5 mL of distilled water and incubating for 30 min, the absorbance was read at 415 nm. A spectrophotometer (Model L800 Aquatic, Germany) was used. The calibration curve was plotted according to Equation (5).

(5) $$y=0.0036x+0.0716;\;R^{2}=0.9987$$

The flavonoid content was reported as mg QE/DW of the sample. Peng et al. (2024) also applied this method for *G. lucidum* [88].

#### 4.8.3. Determination of the Individual Phenolic Compounds by the HPLC-DAD Method

Individual phenolic compounds from *G. lucidum* and *R. pulmo* extracts were analyzed following the methods described by Pesterau et al. (2025) for *R. pulmo* and Veljović et al. (2017) for *G. lucidum* [37,92]. The HPLC method was applied using Agilent 1200 equipment (Agilent Technologies, Santa Clara, CA, USA) with an automatic sampler, a quaternary pump, and a multiple wavelength detector set at 280 nm and 330 nm. A Chem 32 integrator (Agilent) was used to determine retention times (RT) and peak areas. The retention times (± SD, min) of the phenolic standards in the *G. lucidum* extract (0.05 mg/mL) were gallic acid 3.028 ± 0.025, p-coumaric acid 7.19 ± 0.019, kaempferol 1.926 ± 0.024, chlorogenic acid 0.691 ± 0.015, vanillic acid 0.356 ± 0.025, 3-methylgallic acid 0.672 ± 0.008, syringic acid 0.194 ± 0.021, vanillin 0.163 ± 0.051, and ellagic acid 0.112 ± 0.031.

The phenolic compounds in JPC extracted from *R. pulmo* showed the following values for RTs (± SD) of the standards: caftaric acid 0.469 ± 0.05, gallic acid 3.0278 ± 0.025, and syringic acid 0.194 ± 0.03. Individual phenols were determined based on the RT values of the standards. The results were reported as mean ± SD in mg/100 g dry weight (DW) for *G. lucidum* and mg/100 g wet weight (WW) for *R. pulmo.*

### 4.9. Physicochemical Characteristics of the New Biocomposite

#### 4.9.1. Macroscopic and Microscopic Study

A macroscopic study was conducted to evaluate the organoleptic characteristics of both the extracts made from raw materials and the new biocomposite formulations. The *G. lucidum* extracts were prepared in liquid form at different concentrations, while the *R. pulmo* extracts were lyophilized as powders. The biocomposite formulations, E1 (GL-JPC) and E2 (GL-JPC), were prepared by integrating these two biomaterials. For each extract and the final formulations, color and general appearance were recorded.

Microscopic analysis of GL-JPC biocomposites was performed using a Nikon Digital Sight DS-FI2 camera attached to an optical microscope (Nikon Eclipse Imaging System, Tokyo, Japan). Formalin-fixed slides were stained with hematoxylin to facilitate structural observation and assess the uniformity and integrity of the biocomposite components.

#### 4.9.2. Rheological Study

The rheological properties of biocomposite formulations E1 (GL-JPC) and E2 (GL-JPC) were evaluated to determine the rheological behavior of the new biocomposites. Rheological measurements were performed using a Haake VT 550 rheoviscosimeter (Thermo Fisher Scientific, Waltham, MA, USA). The device was equipped with sensors for measuring average viscosity. The data were analyzed using Rheo-Win 4 software. The flow curves and rheograms obtained were used to determine the rheological model corresponding to each formulation. Table 9 shows the calculation Equations (6)–(9) used to determine the rheological parameters.

### 4.10. Antimicrobial Activity

The antimicrobial activity of *G. lucidum* (GL), *R. pulmo* collagen peptides (JPC), and the GL-JPC biocomposite was evaluated against pathogens commonly found in the oral cavity. The following bacteria were studied: Gram-negative bacteria: *P. aeruginosa* (ATCC 27853), *E. coli* (ATCC 25922), and *P. mirabilis* (ATCC 25933); Gram-positive bacteria: *S. mutans* (ATCC 25175), *S. aureus* (ATCC 25923), and the fungal species *C. albicans* (ATCC 10231). The composition of the culture medium is as follows: mix 20 g agar with 3 g beef extract, 5 g peptone, 5 g NaCl in 1 L of distilled water. The culture medium for bacteria was prepared at 37 °C, and a pH of 7.2–7.4 was ensured. To obtain continuous and uniform cultures, the bacterial suspensions were seeded on plates. The antimicrobial activity was determined using a modified Kirby–Bauer diffusion method, and the inhibition zones were measured in triplicate [102]. Ethanol served as a negative control, and amoxicillin as a positive control. Minimum inhibitory concentrations (MICs) were determined using the broth dilution method as described by Tajbakhsh et al. (2011) [139].

Results are reported as the mean ± SD of three independent determinations.

### 4.11. Antioxidant Activity

Antioxidant activity was determined by the DPPH and reducing power assays.

#### 4.11.1. DPPH Method

In assessing antioxidant capacity using the DPPH test, which was performed according to the protocol of Brand et al. (1995) [140]. First, a 1 M DPPH stock solution was prepared. For measurements, 4 mL of DPPH solution was added to 1.5 mL of samples of different concentrations. After 40 min of incubation in the dark, the absorbance was read with a UV-VIS spectrophotometer (A. Kruss UV-VIS 6500, Optronic, Germany) at 517 nm. To obtain the antioxidant activity, we used Equation (10):

(10) $$DPPH\left(\%\right)=\frac{A_{control}-A_{sample}}{A_{control}}\times 100$$

A_control_ and A_sample_ were the absorbance of the control and the analyzed sample, respectively.

The control solution was ascorbic acid. The DPPH test was applied both for *G. lucidum* and *R. pulmo,* but also for the new biocomposite formed by ethanolic extract E2 (GL-JPC). IC50 values were determined using nonlinear regression curves. The antioxidant activity is reported as the mean of three determinations ± SD.

#### 4.11.2. Reducing Power Assay

The antioxidant activity of *G. lucidum*, *R. pulmo*, and biocomposite E2 (GL-JPC) was assessed based on the reducing power assay, which measures their ability to donate electrons and reduce free radicals, as described by Pesterau et al. (2025) and Hsu et al. (2003) [37,141]. The method followed the protocol of Cadar et al. (2019c) [104].

Briefly, 1% potassium ferricyanide phosphate-buffered solution is added to each test sample (1mL) and incubated for 30 min at a warm temperature. The reaction was stopped by adding 2 mL of 10% trichloroacetic acid. Then dilution was performed with double-distilled water, and 2 mL of 0.1% ferric chloride solution was added. Absorbance readings were taken with a UV-VIS spectrophotometer (A. Kruss UV-VIS 6500, Optronic, Germany) at 700 nm. The working standard was ascorbic acid. All samples were tested at identical conditions and concentrations, and the results were reported as the mean ± standard deviation (SD) of three independent determinations.

### 4.12. Biological Evaluation

#### 4.12.1. Cell Viability for SCC-9 and HSC3 Cell Lines

The cytotoxic activity was evaluated in vitro by assessing cell viability on two oral squamous cell carcinoma (OSCC) lines: SCC-9 and HSC3 cells. The experiments were conducted at the Faculty of Dental Medicine, Titus Maiorescu University, Bucharest, Romania, and at the “Sf. Ap. Andrei”, County Clinical Emergency Hospital, Constanța, Romania. The protocol was adapted from Camargo et al. (2022) and Dwiandhono et al. (2020) to suit the current working conditions [31,119].

The cells were centrifuged at 1200 rpm for 10 min, after which fresh DMEM was added to the pellet. After incubating for 20 min, the suspension was centrifuged again under the same conditions. The resulting cell suspension was supplemented with 20% FBS and incubated at 37 °C. The work was carried out in a 5% CO_2_ atmosphere.

Test solutions containing 2 mL of *G. lucidum* (GL) extract, biocomposites E1 (GL-JPC) or E2 (GL-JPC), and 1 mL of DMEM were followed by serial dilutions ranging from 20 to 1000 µg/mL for SCC-9 cells and from 20 to 100 µg/mL for HSC3 cells.

After reaching approximately 80% confluence, the cells were washed with PBS solution and detached using trypsin-EDTA. The detached cells were centrifuged at 1200 rpm for 10 min at 37 °C and seeded into microplates with wells. After 24 h of incubation, MTT reagent was added to the cells at a concentration of 5 mg/mL. The plates were incubated again in the dark at room temperature for 24 h.

Following MTT treatment, cells were exposed to GL, E1 (GL-JPC), or E2 (GL-JPC) at the specified concentrations for 72 h. Absorbance was read at 550 nm using a multimode microplate reader (BioTek 800/TS, Thermo Scientific, Waltham, MA, USA). Low absorbance values corresponded to decreased cell proliferation. Positive control (CTRL^+^) consisted of cells treated with distilled water to induce cell death, while untreated cells (CTRL) were considered 100% viable. All assays were performed in triplicate across *n* = 8 independent experiments, and results were expressed as percentages of cell viability.

#### 4.12.2. Scratch Test on SCC-9 and HSC3 Cell Lines

The scratch assay was performed to evaluate cell migration and wound closure in vitro, following the methods described by Camargo et al. (2022, 2023) with slight modifications [31,62]. SCC-9 and HSC3 cells were seeded in well plates using complete culture medium and incubated for 12 h to allow cell adhesion. After initial incubation, the culture medium was replaced with DMEM/F-12 (Fisher Scientific, Leicestershire, UK) supplemented with 0.5% fetal bovine serum, and cells were incubated for an additional 12 h to reach complete confluence under standard conditions (37 °C, 5% CO_2_). After reaching confluence, a linear scratch was made in the center of each well. The wells were then washed with PBS to remove detached cells, and then the sample was added to each well. Untreated culture medium was used as the control. Cell migration and wound healing were monitored by capturing micrographic images at 0, 24, 48, and 72 h post-scratch using a Nikon Eclipse Ts2R microscope. Images were analyzed with ImageJ software (version 1.54, NIH, Bethesda, MD, USA). The confluence rate, representing the percentage of lesion closure, is expressed as the difference between the closure of the treated area and the total cell-free area immediately after scratching. The results were calculated with Equation (11), as follows:

(11) $$Confluency\;rate\left(\%\right)=\frac{{\mathrm{Area}\;T}_{0}-\mathrm{Area}\;T}{{\mathrm{Area}\;T}_{0}}\times 100$$

where the areas were for the initial times T_0_ and the final time T for each reading.

All assays were performed in triplicate for *n* = 8 independent experiments with distinct cell passages and were analyzed for each wound.

### 4.13. Statistical Methods

Statistical analysis of experimental data was performed using SPSS 16.0 software. The test parameters applied were those used by one-way ANOVA, Student’s *t*-test, and Duncan’s multiple range test to evaluate differences in the chemical composition, properties, and characteristics of the studied samples (GL, E1 (GL-JPC), and E2 (GL-JPC). Statistical significance was set at *p* < 0.05 for most analyses, and *p* < 0.01 for selected parameters.

## 5. Conclusions

In the actual context, products from natural sources represent a promising research direction based on their rich composition of bioactive compounds with antitumor, antioxidant, and antimicrobial properties. This study constitutes a preliminary investigation, the first stage in evaluating a new formulation for oral cancer treatment, and is part of a broader project focused on developing biomaterials from natural extracts for therapeutic applications. The innovative aspect of this research lies in the development of a novel biocomposite combining two natural sources: the fungal extract from *G. lucidum* and the marine collagen extract from *R. pulmo*. This combination demonstrated promising in vitro anticancer activity. The research emphasizes thorough physicochemical characterization of each component, as well as the final composites (E1 (GL-JPC) and E2 (GL-JPC)), which evidenced their rheological properties, bioactive profiles, and key compounds such as amino acids, phenolics, and polysaccharides, which collectively contribute to the observed antitumor, antioxidant, and antimicrobial activities of the new biocomposite.

In vitro evaluation on oral cavity tumor cells demonstrated that the formulations GL, E1 (GL-JPC), and E2 (GL-JPC) induced a significant dose-dependent reduction in cell viability. These results support the hypothesis that polysaccharides, polyphenols, and other bioactive compounds such as terpenes from *G. lucidum*, combined with collagen peptides from *R. pulmo*, exert a synergistic effect with notable antitumor activity. However, further in vitro studies on additional tumor cell lines, as well as in vivo investigations, are necessary completely explicate the pharmacokinetics, mechanisms of action, biodegradation processes, and cellular interactions of the new formulation. For future practical applications, extensive studies are required to address potential uncertainties related to extraction efficiency, structural variability, and cytotoxicity, which could affect the development of novel therapeutic solutions. Moreover, coordinated strategies at the European level are crucial to support sustainable jellyfish harvesting and cultivation of medicinal *G. lucidum* as sources of natural bioactive compounds for biomedical applications. Optimizing extraction processes, ensuring reproducibility, and developing sterile and stable biocomposite formulations are fundamental steps for future clinical applicability.

In conclusion, fungal extracts and marine collagen from jellyfish represent a promising direction for the development of innovative complementary oncological therapies. These biocomposites, through the synergistic action of their active biocompounds, provide a strong foundation for future in vivo studies and for the validation of their antitumor potential.

## Figures and Tables

**Table 1:** Chemical composition of ethanolic fungal extract and collagen peptides hydrogels.

Characteristics	70% Ethanolic Extract of *G. lucidum*		Hydrogel Extract JPC from *R. pulmo*	
	*G. lucidum*	References	With 10% Pepsin	References
Moisture % (DW)	11.98 ± 4.34	12.19 ± 0.33 [46] 8.14 ± 1.12 [48]	13.1 ± 0.10	-
Ash 600–800 °C % (DW)	2.84 ± 0.50	3.93 ± 0.18 [46] 2.4 ± 0.20 [49]	0.55 ± 0.10	-
Polysaccharides % (DW)	42.80 ± 5.53	44.19 ± 0.17 [46] 37.33 ± 4.06 [48] 88.4 ± 0.20 [49]	1.25 ± 0.30 W; 0.65 ± 0.25 G	-
Proteins % (DW)	7.49 ± 0.56	8.54 ± 0.87 [48] 6.72 ± 0.04 [49] 19.5 ± 0.04 [50]	59.48 ± 0.55 23.29 ± 0.85 B; 20 ± 1.21 OA; 17.07 ± 1.85 G	61.80 [51] 6 W; 8.70–13.70 B; [53] 27 OA; 18 G [52]
Collagen content % (DW)	-	-	55.5 ± 0.60	56.3 [53]; 57.1 [37]
Lipids % (DW)	2.83 ± 0.18	2.4 ± 0.29 [46] 2.50 ± 0.02 [50] 3.00 ± 0.01 [50]	5.20 ± 0.79 W; 1.55 ± 0.10 G	2.3 W; [53] 4.0 ± 0.10 W; 0.80 OA; 1.20 G; [53]
Total dietary fiber % (DW)	2.90 ± 0.01	3.5 ± 0.01 [50]	-	-

(W)—the whole body; (B)—bell; (OA)—oral arms; and (G)—gonads. Values are expressed as mean ± SD for *n* = 3.

**Table 2:** Types of monosaccharides that form the polysaccharide content in the aqueous extract from the fruiting body of *G. lucidum*.

Monosaccharide Structures	Aqueous Extract of *G. lucidum* (mg/g)
Xylose	0.41 ± 0.01
Fructose	14.50 ± 0.02
Glucose	12.91 ± 0.02
Sucrose	0.82 ± 0.01
Maltose	51.01 ± 0.01

Results are expressed as mean ± SD for three replicates (*n* = 3).

**Table 3:** Interpretation of FTIR spectrum signals of collagen in *R. pulmo*.

Region	Wavelength	Allocation	Wavelength from References
Aminde A	3305.75 cm^−1^	N-H stretch coupled with a hydrogen bond	3.283cm^−1^ [56]
Amide B	2929.41 cm^−1^	CH_2_ asymmetric stretching	2.934 cm^−1^ [56]
Amide I	1651.60 cm^−1^	Stretching C=O/hydrogen bond coupled with COO^−^	1.647 cm^−1^ [39]
Amide II	1539.31 cm^−1^	NH bending coupled with CN stretching, CH_2_ bending, COO^−^ symmetric stretching, CH_2,_ hydrogen bonds.	1.550 cm^−1^ [55]
Amide III	1241.18 cm^−1^	NH bending coupled with CN stretching, C-O stretching	1.238 cm^−1^ [56]

**Table 4:** The results for amino acid content.

Amino Acid Type	*R. pulmo* from Black Sea Coast (Residues/1000 Residues)	*R. pulmo* from Goa Coast, India (%) [57]
Essential amino acids (EAAs)		
Cystine (*Cys*)	1.20	-
Arginine (*Arg*)	6.20	5.63
Glycine (*Gly*)	33.40	29.34
Glutamic acid (*Glu*)	15.20	13.46
Leucine (*Leu*)	8.60	6.35
Histidine (*His*)	0.60	-
Proline (*Pro*)	3.90	2.97
Lysine (*Lys*)	6.30	4.62
Threonine (*Thr*)	5.25	3.18
Hydroxiproline (*Hyp*)	3.65	4.82
Valine (*Val*)	4.90	2.80
Tyrosine (*Tyr*)	3.90	1.77
Triptophan (*Trp*)	2.80	4.72
Non-essential aminoacids (NEAAs)		
Aspartic acid (*Asp*)	6.65	10.91
Alanine (*Ala*)	6.90	10.38
Serine (*Ser*)	1.70	-

**Table 5:** Total phenolic and flavonoid content of *G. lucidum* and *R. pulmo*.

TPC				TFC	
*G. lucidum* (mg GAE/g DW)		*R. pulmo* (μg GAE/g DW)		*G. lucidum* (mg QE/g DW)	
35.80 ± 0.50	30.916 ± 0.50 [46]	6.58 ± 0.50	2.07 ± 0.50 [50]	65.32 ± 0.50	34.09–38.08 [57]

Data are expressed by Mean ± SD for *n* = 3.

**Table 6:** Individual phenolic content of *G. lucidum* and *R. pulmo*.

Type	Results for GL mg/100 g DW	Results for JPC mg/100 g WW
p-coumaric Acid	0.038 ± 0.02	-
Vanillic Acid	0.10 ± 0.01	-
Gallic Acid	742.08 ± 0.02	5.84 ± 0.02
Chlorogenic Acid	0.12 ± 0.01	-
Kaempferol	59.57 ± 0.01	-
Vanillin	0.10 ± 0.02	-
Ellagic Acid	3.78 ± 0.02	-
3-Methil-Gallic Acid	1.56 ± 0.02	-
Syringic acid	0.40 ± 0.01	0.50 ± 0.009
Caftaric acid	-	0.24 ± 0.01

Data are expressed by Mean ± SD for *n* = 3.

**Table 7:** Characteristics of ingredients for the newly formulated biocomposite.

Compound	Color	Observation
*G. lucidum* 60% ethanolic extract	yellow-brown	Liquid
*G. lucidum* 75% ethanolic extract	brown	Liquid
Collagen peptides from *R. pulmo*	white	Powder
Hydrogel with 3/5 parts of extract of ethanolic GL 10% and 2/5 parts extract of *R. pulmo* collagen peptide extract (E1)	light yellow	viscous, gelatinous texture
Hydrogel with 4/5 parts of 10% ethanolic GL extract and 1/5 parts of *R. pulmo* collagen peptide extract (E2)	light brown	viscous, gelatinous texture

**Table 8:** Minimum Inhibitory Concentration (MIC) of the extract and the newly formulated composite.

Bacterial Strain	MIC (μg/mL)		
	*G. lucidum*	*R. pulmo*	GL-JPC Composite
*S. mutans*	85 ± 0.7	50 ± 0.5	86.5 ± 0.8
*S. aureus*	80 ± 0.5	75 ± 0.4	85 ± 0.5
*C. albicans*	75 ± 0.6	75 ± 0.2	75 ± 0.8
*E. coli*	76 ± 0.8	75 ± 0.3	77 ± 0.5
*P. aeruginosa*	50 ± 0.8	50 ± 0.6	52 ± 0.6
*P. mirabilis*	35 ± 0.8	25 ± 0.5	36 ± 0.5

Results are expressed as mean ± SD for *n* = 3.

**Table 9:** Rheological parameters and equations.

Viscosity ɳ (cP) Depending on Shear Speed D (s^−1^)	Shear Speed D (s^−1^) in Correlation with the Selected Rotation Speed ω (rpm)	Shear Speed D (s^−1^) Depending on Shear Stress τ (Pa)	Shear Stress τ (Pa) Depending on Viscosity ɳ (cP) and Shear Speed D (s^−1^)
ɳ = f(D)	D = ω × R	D = f(τ)	Τ = ɳ × D
(6)	(7)	(8)	(9)

## Data Availability

The raw data supporting the conclusions of this article will be made available by the authors on request.

## Abbreviations

The following abbreviations are used in this manuscript:

GL	*Ganoderma lucidum*
HWE	Hot Water Extract
EDTA-Na	Ethylene Diamine Tetra acetic Acid Sodium Salt
BSA	Bovine Serum Albumin
CD	Circular Dichroism
HPLC	High-Performance Liquid Chromatography
RT	Retention Time
AOAC	Association of Official Analytical Chemists
JPC	Jellyfish-derived Collagen Peptides
SDS-PAGE	Sodium Dodecyl Sulfate–Polyacrylamide Gel Electrophoresis
FT-IR	Fourier-Transform Infrared Spectroscopy
PSC	Pepsin-Soluble Collagen
TPC	Total Phenolic Compounds
TFC	Total Flavonoid Content
GAE	Gallic Acid
QE	Quercetin
DPPH	α-Difenil-β-Picrilhidrazil
MIC	Minimum Inhibitory Concentration
MTT	3-(4,5-dimethylthiazol-2-yl)-2,5-diphenyltetrazolium bromide
DMEM	Dulbecco’s Modified Eagle Medium
FBS	Fetal Bovine Serum
OSCC	Oral Squamous Cancer Cells
